# Decreasing the electronic confinement in layered perovskites through intercalation†
†Electronic supplementary information (ESI) available. CCDC 1487885. For ESI and crystallographic data in CIF or other electronic format see DOI: 10.1039/c6sc02848a



**DOI:** 10.1039/c6sc02848a

**Published:** 2016-11-10

**Authors:** Matthew D. Smith, Laurent Pedesseau, Mikaël Kepenekian, Ian C. Smith, Claudine Katan, Jacky Even, Hemamala I. Karunadasa

**Affiliations:** a Department of Chemistry , Stanford University , Stanford , CA 94305 , USA . Email: hemamala@stanford.edu; b Fonctions Optiques pour les Technologies de l'information , CNRS , INSA de Rennes , 35708 Rennes , France . Email: Jacky.Even@insa-rennes.fr; c Institut des Sciences Chimiques de Rennes , CNRS , Université de Rennes 1 , 35042 Rennes , France

## Abstract

We show that post-synthetic small-molecule intercalation can significantly reduce the electronic confinement of 2D hybrid perovskites. Using a combined experimental and theoretical approach, we explain structural, optical, and electronic effects of intercalating highly polarizable molecules in layered perovskites designed to stabilize the intercalants. Polarizable molecules in the organic layers substantially alter the optical and electronic properties of the inorganic layers. By calculating the spatially resolved dielectric profiles of the organic and inorganic layers within the hybrid structure, we show that the intercalants afford organic layers that are more polarizable than the inorganic layers. This strategy reduces the confinement of excitons generated in the inorganic layers and affords the lowest exciton binding energy for an *n* = 1 perovskite of which we are aware. We also demonstrate a method for computationally evaluating the exciton's binding energy by solving the Bethe–Salpeter equation for the exciton, which includes an *ab initio* determination of the material's dielectric profile across organic and inorganic layers. This new semi-empirical method goes beyond the imprecise phenomenological approximation of abrupt dielectric-constant changes at the organic–inorganic interfaces. This work shows that incorporation of polarizable molecules in the organic layers, through intercalation or covalent attachment, is a viable strategy for tuning 2D perovskites towards mimicking the reduced electronic confinement and isotropic light absorption of 3D perovskites while maintaining the greater synthetic tunability of the layered architecture.

## Abstract 2

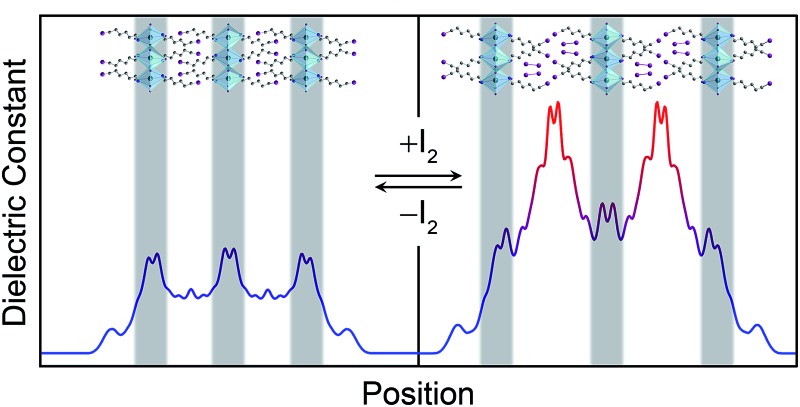
We show that post-synthetic small-molecule intercalation can significantly reduce the electronic confinement of 2D hybrid perovskites.

## 1. Introduction

The remarkable optoelectronic properties of organic–inorganic metal–halide perovskites have recently come to light. Notably, these properties can be synthetically modulated by altering the dimensionality of the anionic inorganic lattice. Small organic cations allow for the self-assembly of three-dimensional (3D) frameworks, whereas larger organic cations partition the inorganic lattice into two-dimensional (2D) sheets.^1^ Recently, 3D Pb–I perovskites have been employed as low-cost absorbers in high-efficiency solar cells.^2–4^ Here, the weak binding energy between photogenerated electrons and holes allows for charge carriers to easily separate and migrate towards their respective current collectors. The high dielectric constant of the 3D Pb–I lattice shields the coulombic attraction between photogenerated electrons and holes.^5,6^ This results in free charge carriers that are ideal for solar-cell absorbers. On the other hand, typical 2D *n* = 1 perovskites (where *n* is the number of metal–halide sheets in each inorganic layer) are not suitable for standard photovoltaic devices, owing to the presence of strongly bound electron–hole pairs, or excitons. The quantum confinement of the 2D inorganic sheets increases the bandgap (*E*_g_) and exciton binding energy (*E*_b_) relative to the 3D materials. The *E*_b_ in these materials is further enhanced by the dielectric mismatch between organic and inorganic layers.^7–12^ Here, the low dielectric constant of the adjacent organic layers provides poor shielding of the electrons and holes in the inorganic layers leading to the exciton's dielectric confinement. The *E*_b_ values for *n* = 1 perovskites are typically above 300 meV,^13^ comparable to those of certain organic molecules.^14^ These tightly bound excitons in 2D perovskites have enabled varied applications in luminescence. The high oscillator strength of the excitons affords strong excitonic luminescence, which has been used in green phosphor^15^ and light-emitting-diode^16^ applications, and we proposed that white-light emission from 2D perovskites stems from strongly bound excitons stabilized (or self-trapped) through lattice distortions.^17,18^ Furthermore, perovskites with 1 < *n* < ∞ values provide access to intermediate *E*_g_ and *E*_b_ values,^19–21^ while the organic layers bring new functionality. For example, the *n* = 3 2D Pb–I perovskite exhibits sufficiently low *E*_g_ and *E*_b_ values to absorb sunlight and generate photocurrent in a solar cell, while hydrophobic organic layers provide enhanced moisture resistance.^22,23^ Owing to the greater stability and structural diversity of the layered framework compared to the 3D perovskite, we investigated how the common *n* = 1 Pb–I perovskites can be modified to mimic the optoelectronic properties of their 3D congeners.

Herein we demonstrate that post-synthetic intercalation of highly polarizable molecules into 2D Pb–I perovskites can significantly decrease their electronic confinement and optical anisotropy, enabling new functionality in these materials. Using experimental and theoretical methods, we explain the structural, optical, and electronic consequences of intercalating I_2_ molecules in the perovskite's organic layers. Furthermore, using *in situ* powder X-ray diffraction (PXRD) and *in situ* optical absorption spectroscopy, we monitor the dynamics of halogen intercalation and release. We further extend halogen intercalation to halogen-mediated reactivity to access novel Pb–halide perovskites that cannot be synthesized from solution.

## 2. Results and discussion

We recently showed that upon exposure to halogen gas, layered perovskites containing terminal alkynes or alkenes topotactically expand by up to 36% of their original volume to form perovskites containing dihaloalkenes or dihaloalkanes, respectively.^24,25^ During our experiments with I_2_ chemisorption, we hypothesized that intercalation precedes halogenation of these nonporous solids. Here, the organic layers may stabilize I_2_ incorporation similar to halogen solvation in organic liquids. A paraffin-like quality has been previously ascribed to organic layers containing long-chain alkyl groups in hybrid perovskites^26,27^ and binding sites in the organic layers have been shown to stabilize electrochemical ion insertion in these materials.^28^ We therefore sought to assess if intercalation of highly polarizable molecules could provide a facile method for tuning the electronic structure of 2D perovskites.

### 2.1. Structural effects of iodine intercalation

**Fig. 1:**
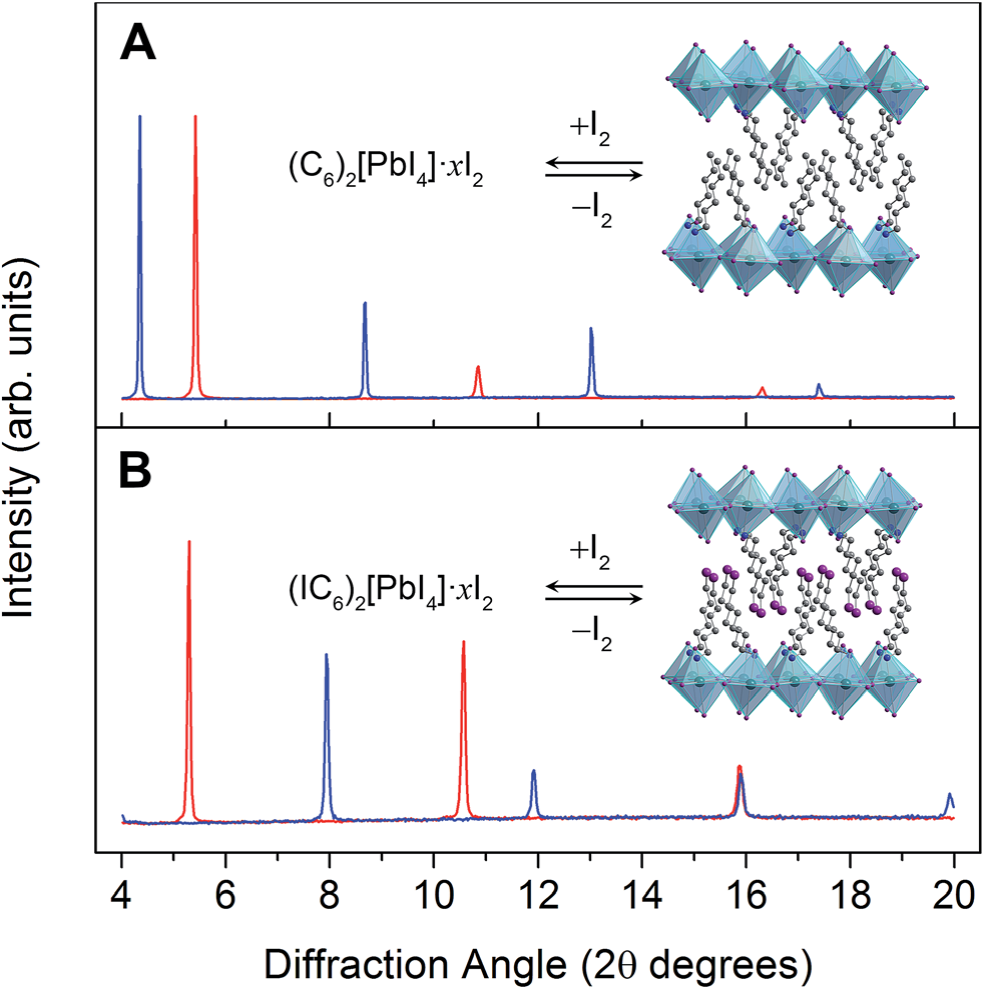
(A) Powder X-ray diffraction (PXRD) patterns of (C_6_)_2_[PbI_4_] (red) and (C_6_)_2_[PbI_4_]·*x*I_2_ (blue). Inset: X-ray crystal structure of (C_6_)_2_[PbI_4_].^30^ (B) PXRD patterns of (IC_6_)_2_[PbI_4_] (red) and (IC_6_)_2_[PbI_4_]·*x*I_2_ (blue). Inset: X-ray crystal structure of (IC_6_)_2_[PbI_4_]. Dark green, purple, blue, and grey spheres represent Pb, I, N, and C atoms, respectively. Disordered atoms and hydrogen atoms are omitted for clarity.

Weak electrostatic interactions have been previously leveraged to reversibly intercalate nonpolar aliphatic and aromatic hydrocarbons into layered perovskites containing long alkyl chains such as (C_10_H_21_NH_3_)_2_[CdCl_4_] and (C_9_H_19_NH_3_)_2_[PbI_4_].^27^ Stronger fluoroaryl–aryl interactions have been subsequently used to stabilize and isolate the guest-intercalated perovskites (C_6_H_5_(CH_2_)_2_NH_3_)_2_[SnI_4_]·(C_6_F_6_) and (C_6_F_5_(CH_2_)_2_NH_3_)_2_[SnI_4_]·(C_6_H_6_).^29^ In order to investigate the consequences of I_2_ intercalation in 2D perovskites, we first chose (C_6_H_13_NH_3_)_2_[PbI_4_]^30^ (hereafter denoted as (C_6_)_2_[PbI_4_]) in order to provide a well-defined organic bilayer with some structural flexibility (Fig. 1A, inset). Perovskite films were deposited on substrates through spin coating and then thermally annealed. Flowing air over the spinning sample improved film quality (further details are provided in the ESI†). Exposing a (C_6_)_2_[PbI_4_] film to a dry, inert carrier gas (N_2_ or Ar) containing I_2_ vapor results in a color change from orange to red and an expansion of the unit cell by *ca.* 4 Å along the *c* axis (perpendicular to the inorganic sheets) to yield the perovskite we formulate as (C_6_H_13_NH_3_)_2_[PbI_4_]·*x*I_2_ (hereafter (C_6_)_2_[PbI_4_]·*x*I_2_). However, (C_6_)_2_[PbI_4_]·*x*I_2_ completely desorbs I_2_ within 10 minutes of removal from an I_2_ atmosphere to regenerate the original material (C_6_)_2_[PbI_4_].

**Fig. 2:**
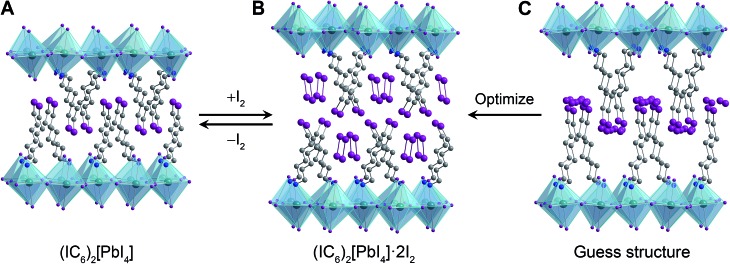
(A) Single-crystal X-ray structure of (IC_6_)_2_[PbI_4_]. Initial guess structure of the I_2_ intercalation product (C) and its geometry-optimized structure (B). Dark green, purple, blue, and grey spheres represent Pb, I, N, and C atoms, respectively. Disordered atoms and hydrogen atoms are omitted for clarity.

To stabilize the I_2_-intercalated material, we then focused on the perovskite (IC_6_H_12_NH_3_)_2_[PbI_4_] (hereafter denoted as (IC_6_)_2_[PbI_4_]) containing terminal alkyl iodides (Fig. 1B, inset). The structure of (IC_6_)_2_[PbI_4_] at 173 K has been reported;^31^ however, we collected single-crystal XRD data at 298 K to gain a better match with our room-temperature PXRD patterns. The 298 K crystal structure (Fig. 2A) shows a partially interdigitated organic layer with I···I distances of 5.017(1) Å between organoiodines that run parallel to the inorganic sheets. Iodine–iodide interactions of 3.955(1) Å are also evident between the organoiodines and terminal iodides of the inorganic sheets. We envisioned that halogen–halogen interactions^32,33^ between the organoiodines and inorganic iodides could stabilize I_2_ intercalation in the organic layers. Exposing (IC_6_)_2_[PbI_4_] thin films to I_2_ vapor also results in a color change from orange to red. PXRD data show that I_2_ exposure causes the inter-layer spacing in (IC_6_)_2_[PbI_4_] to increase by 5.5 Å (33%) (Fig. 1B). Indeed, the iodine retention time is increased more than four-fold in (IC_6_)_2_[PbI_4_]·*x*I_2_ compared to (C_6_)_2_[PbI_4_]·*x*I_2_. Because films of (C_6_)_2_[PbI_4_] and (IC_6_)_2_[PbI_4_] are similar in both quality and thickness (250 ± 20 nm), we attribute the difference in I_2_ retention to stabilization of I_2_ molecules by the iodoalkyl groups in (IC_6_)_2_[PbI_4_].

To elucidate the structure of (IC_6_)_2_[PbI_4_]·*x*I_2_, we performed structural optimization at the DFT level in two steps. First, we used the local density approximation (LDA) for both (IC_6_)_2_[PbI_4_] and (IC_6_)_2_[PbI_4_]·*x*I_2_. Then, we re-optimized the LDA-optimized structure using the generalized gradient approximation (GGA) corrected to take into account van der Waals (vdW) interactions (hereafter GGA+vdW; see ESI and Table S2† for details). The alkyl chain of the IC_6_^+^ cation in (IC_6_)_2_[PbI_4_] is disordered across two positions. For the calculations we used only the atomic positions of the major disorder component. This final geometry-optimized structure of (IC_6_)_2_[PbI_4_] is consistent with the room-temperature single-crystal XRD structure, with differences ranging from *ca.* 1.5% to 3.8% for the *a* and *b* lattice parameters, respectively (Table S2†). Note that our structure optimizations do not include temperature effects. In layered hybrid perovskites, the evolution of unit-cell parameters with temperature is not necessarily systematic because of the rotational flexibility of the metal–halide octahedra.

In order to simulate the I_2_-intercalated perovskite structure, we expanded the inter-layer spacing by the experimentally observed 5.5 Å. We then positioned two intercalated I_2_ molecules per formula unit with their inter-nuclear axes aligned parallel to the inorganic sheets to simulate their possible configuration as the molecules enter the perovskite (Fig. 2C). During geometry optimization, no constraints were placed upon the added iodine atoms, which were allowed to move freely and independently. Upon reaching an optimized configuration at the LDA level of theory, we continued the optimization at the GGA+vdW level of theory. We find that the iodine atoms arrange as I_2_ molecules with their inter-nuclear axes nearly perpendicular to the inorganic sheets (Fig. 2B). The I_2_ molecules are flanked on either side by halogen–halogen interactions from both inorganic iodides (in the inorganic layer) as well as organoiodines (in the organic layer) with short I···I distances of 3.32–3.33 Å (Table S2†). The computed *c* lattice parameter (double the inter-layer spacing) for (IC_6_)_2_[PbI_4_]·2I_2_ compares well with that obtained from PXRD, with a difference of *ca.* 1% (Table S2†) of the experimental value determined at room temperature. We therefore assign the I_2_-intercalated perovskite as (IC_6_)_2_[PbI_4_]·2I_2_. Interestingly, the I_2_···I^–^ fragment in (IC_6_)_2_[PbI_4_]·2I_2_ exhibits a similar geometry to the triiodide anion found in CsI_3_,^34^ indicating that the inorganic lattice may now be thought to contain axial triiodides bound to the Pb^2+^ centers to give the perovskite (IC_6_)_2_[PbI_2_(I_3_)_2_]. Therefore, we post-synthetically access a lead–iodide–triiodide perovskite, which is structurally related to the mixed-ligand 2D perovskites (H_3_N(CH_2_)_8_NH_3_)_2_[(Au^I^I_2_)(Au^III^I_4_)(I_3_)_2_]^35^ and (CH_3_NH_3_)_2_[PbI_2_(SCN)_2_]^36^ and the 1D structure (H_3_N(CH_2_)_2_SS(CH_2_)_2_NH_3_)_4_[Pb_3_I_14_]·I_2_ (ref. 37) that contains I_2_ molecules interacting with the lead–iodide chains.

### 2.2. Optical and electronic effects of iodine intercalation

**Fig. 3:**
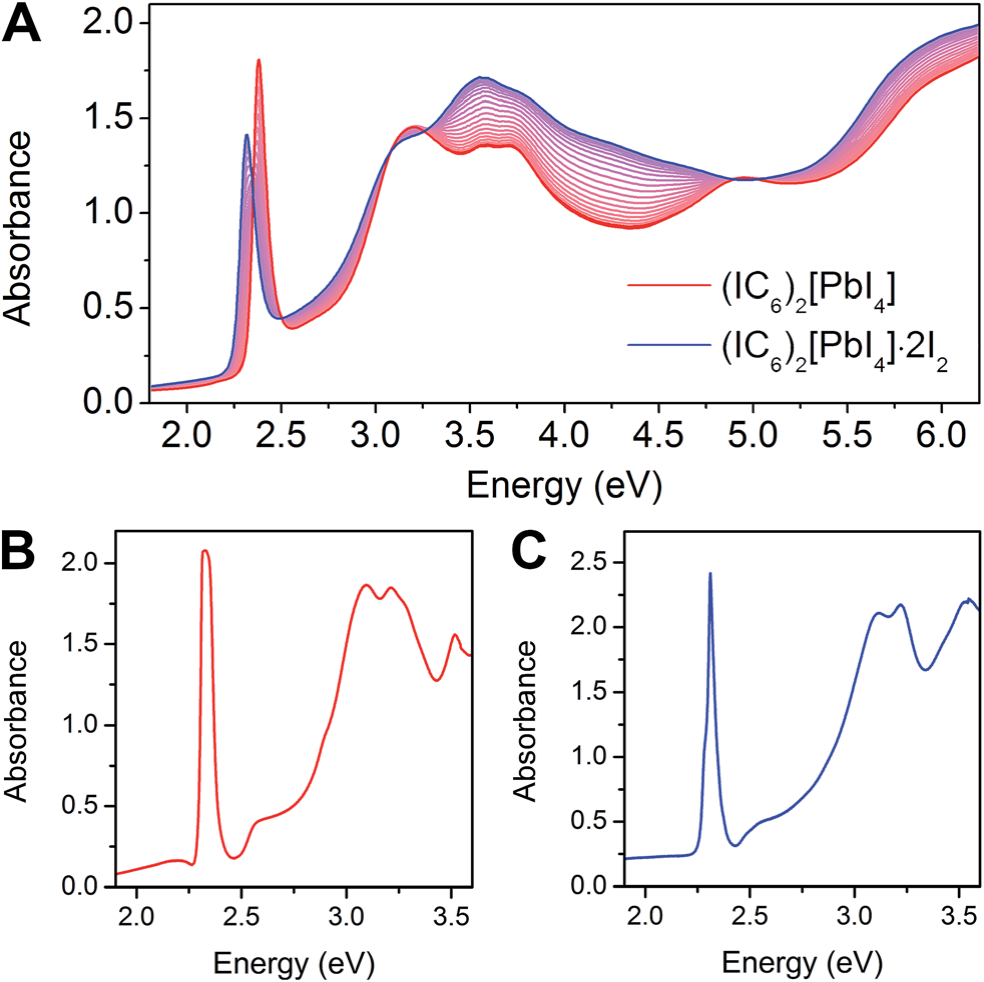
(A) Change in room-temperature absorption spectra as (IC_6_)_2_[PbI_4_] (red) absorbs I_2_ to form (IC_6_)_2_[PbI_4_]·2I_2_ (blue). Absorbance spectra collected at 5 K for (IC_6_)_2_[PbI_4_] (B) and (IC_6_)_2_[PbI_4_]·2I_2_ (C).

The 2D Pb–I perovskites are medium-bandgap semiconductors with significant absorption in the visible range. The 298 K absorption spectrum of (IC_6_)_2_[PbI_4_] shows an excitonic absorption at 2.38 eV (521 nm), similar to other layered Pb–I perovskites (Fig. 3A).^38^ Iodine intercalation causes a redshift of this absorption band by 60 meV to 2.32 eV (535 nm), as well as an increase in absorption between 3 and 5 eV. Furthermore, the intensity ratio between the excitonic absorption peak and the above bandgap (continuum) absorption is reduced upon I_2_ intercalation. The high oscillator strength and temperature-induced broadening of the excitonic absorbance in lead–halide perovskites^13,38^ obscure the bandgap onset at room temperature. However, at 5 K we were able to resolve the bandgap in both (IC_6_)_2_[PbI_4_] and (IC_6_)_2_[PbI_4_]·2I_2_ as linear step-like absorption features (Fig. 3B and C and S2†) characteristic of a 2D material.^39^ We estimate *E*_g_ for (IC_6_)_2_[PbI_4_] to be *ca.* 2.56 eV. Iodine intercalation redshifts the *E*_g_ to *ca.* 2.49 eV in (IC_6_)_2_[PbI_4_]·2I_2_, a decrease of *ca.* 70 meV. Importantly, the perovskite's *E*_b_ substantially decreases upon I_2_ intercalation. Using the difference between the bandgap and the exciton peak energy in the 5 K absorption spectra, we estimate that *E*_b_ for (IC_6_)_2_[PbI_4_] is *ca.* 230 meV, which is significantly lower than typical perovskites likely owing to the polarizability of the organoiodines. This *E*_b_ value further drops in (IC_6_)_2_[PbI_4_]·2I_2_ to only 180 meV (a decrease of 50 meV). To our knowledge, this is the lowest *E*_b_ value reported for an *n* = 1 Pb–I perovskite. Most 2D lead–iodide perovskites have *E*_b_ values higher than 300 meV.^7^ The perovskite (C_6_H_5_(CH_2_)_2_NH_3_)_2_[PbI_4_] has been reported to have a notably low *E*_b_ of 220 meV, attributed to the polarizability of the aromatic organic cations.^21^

Small-molecule intercalation can cause both structural and electronic changes in perovskites. As the inorganic sheets are pushed apart to accommodate the intercalants, small distortions in the metal–halide sheets can slightly alter *E*_g_.^40^ However, this effect alone cannot explain the significant reduction in both *E*_b_ and *E*_g_ we observe upon I_2_ intercalation. In fact, the computed crystal structure reveals a sizeable increase of in-plane octahedral rotations (Table S2†) that should lead to an increase in *E*_g_,^41–43^ in contradiction with our experimental findings. Therefore, intercalation-induced electronic effects should play the dominant role in the observed changes in the perovskite.

Two cooperative electronic effects greatly modify the *E*_b_ of 2D perovskites relative to the 3D analogues: (i) quantum and (ii) dielectric confinement.^13,38^ (i) Quantum confinement arises from the 2D structure of the lead–halide layers, causing greatly reduced band dispersion perpendicular to the inorganic sheets.^38,39^ In the ideal limit of a single *n* = 1 lead–halide layer confined by potential barriers of infinite height, purely 2D quantum confinement enhances *E*_b_ by a factor of four compared to the analogous 3D perovskite.^39,44^ Therefore, to parse the contribution of quantum confinement to the 2D perovskite's *E*_b_, we first considered a hypothetical 3D perovskite with the same dielectric constant and exciton reduced mass as the 2D perovskite. Here, the 3D material's *E*_b_ can be calculated as the Rydberg energy in the hydrogenic Bohr model modified by the dielectric constant of the inorganic layers (measured at the middle of an inorganic sheet in the 2D perovskite, close to the Pb atoms) and exciton reduced mass (details in the ESI†). Using our computed values of the 3D Rydberg energies of the excitons *E*_b,3D_, and calculating *E*_b,2D_ = 4 *E*_b,3D_ gives values of 204 and 142 meV for (IC_6_)_2_[PbI_4_] and (IC_6_)_2_[PbI_4_]·2I_2_, respectively. These values are lower than the corresponding experimental values (230 and 180 meV, respectively), indicating significant additional contributions from the exciton's dielectric confinement to its *E*_b_. (ii) Dielectric confinement^11^ is a result of the dielectric mismatch between the inorganic (high dielectric constant)^5,45^ and organic (low dielectric constant)^38^ layers. Here, the high-frequency dielectric constant (*ε*_∞_) is an appropriate descriptor of the charge-screening ability of the layers, owing to the small exciton Bohr radius and faster timescales of electronic polarization compared to lattice vibrations.^5,46^ The organic layer poorly screens the electric field between the electron and hole in the inorganic layer, thereby further enhancing *E*_b_ over the conventional limit of a 2D quantum well.^9,13^

We reasoned that I_2_ intercalation could lessen the quantum confinement (the I_2_ molecules that orient perpendicular to the sheets could reduce its 2D nature), reduce the dielectric confinement (by increasing the polarizability of the organic layers), or both. To parse out these contributions to the exciton's confinement we turned to electronic-structure calculations.

#### Electronic structure

**Fig. 4:**
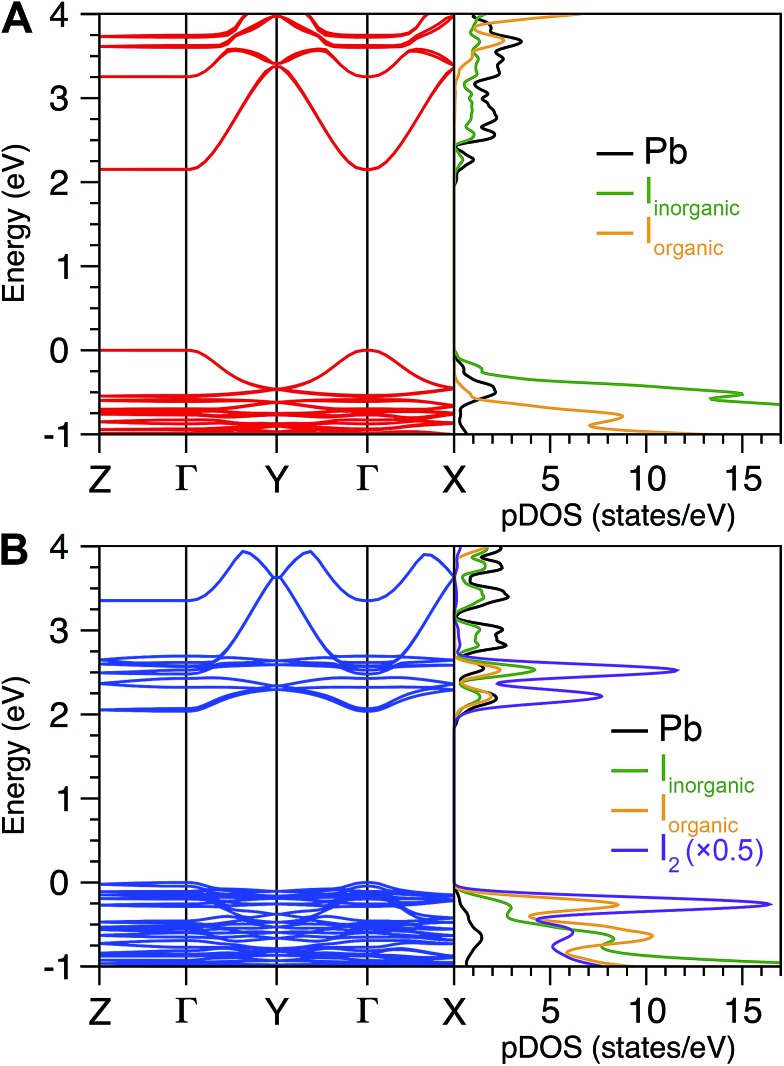
Electronic band structure (left) and projected density of states (pDOS, right) for (A) (IC_6_)_2_[PbI_4_] and (B) (IC_6_)_2_[PbI_4_]·2I_2_. The I_2_ pDOS was halved to bring into scale.

Starting from the geometry-optimized structures for (IC_6_)_2_[PbI_4_] and (IC_6_)_2_[PbI_4_]·2I_2_ (optimized at the GGA+vdW level of theory), we investigated the electronic consequences of I_2_ intercalation. Since this level of theory cannot accurately calculate semiconductor bandgaps, we used the Heyd, Scuseria, and Ernzerhof (HSE) hybrid functional.^47–49^ This functional is known to improve accuracy without reaching the performance of GW calculations that remain computationally unaffordable for this type of large, low-symmetry system. We also included spin–orbit coupling (SOC), which is essential for correctly evaluating the band structures of lead–halide perovskites (Table S3 and Fig. S3†).^12^ The HSE+SOC band structure of (IC_6_)_2_[PbI_4_] (Fig. 4A) shows a direct bandgap at the Brillouin zone center with an *E*_g_ of 2.15 eV. Consistent with prior reports, hybridization of the I 5p and Pb 6s orbitals leads to a large in-plane dispersion of the valence-band (VB) maximum, while the Pb 6p orbitals provide for the in-plane dispersion of the conduction-band (CB) minimum.^12,50^ The negligible band dispersion along the *Γ*–*Z* direction indicates the 2D nature of the electronic structure responsible for quantum confinement. A similar VB and CB composition is found for (IC_6_)_2_[PbI_4_]·2I_2_ (Fig. 4B) with a direct bandgap at *Γ*. The calculated *E*_g_ decreases by 110 meV upon I_2_ intercalation. Including self-energy corrections that account for interactions of charge carriers with the potential of the surrounding medium (including dielectric effects), the calculated decrease in *E*_g_ upon intercalation is 230 meV (details in the ESI†). In contrast to the band structure of (IC_6_)_2_[PbI_4_], a set of narrow bands appears close to the Fermi level for the I_2_-intercalated perovskite. From the projected density of states (pDOS), we see that these originate from the guest I_2_ molecules (Fig. 4). The highest-energy valence bands stemming from the inorganic sheets lie about 200 meV below the Fermi level. Solid (IC_6_)_2_[PbI_4_]·2I_2_ can be regarded as a weakly coupled composite system with limited interaction between the narrow bands arising from the intercalated I_2_ molecules and the original energy levels of (IC_6_)_2_[PbI_4_]. The bands derived from the intercalated I_2_ molecules decrease the dispersion of the original CB minimum by *ca.* 30%, although the dispersion of the original VB maximum is mostly unaffected.

#### Reduced optical anisotropy

We computed the frequency-dependent dielectric matrix elements in order to calculate the perovskites' absorption spectra. Fig. S4† shows absorbance spectra calculated for (IC_6_)_2_[PbI_4_] and (IC_6_)_2_[PbI_4_]·2I_2_. Absorption of light propagating perpendicular to the inorganic sheets (given by the imaginary component of the frequency-dependent dielectric constant parallel to the inorganic sheets *ε*′′_∥_) remains nearly unaffected by the presence of I_2_. However, absorption of light propagating parallel to the inorganic sheets (given by the imaginary component of the frequency-dependent dielectric constant perpendicular to the inorganic sheets *ε*′′_⊥_) is dramatically altered upon I_2_ insertion. Pristine (IC_6_)_2_[PbI_4_] shows a strongly anisotropic optical response with minimal absorbance of low-energy light incident parallel to the inorganic sheets, which is characteristic of 2D materials with no electronic communication between layers.^12^ Upon I_2_ intercalation, we see a large decrease in this anisotropy. This likely causes the new above-gap absorption features we observe upon I_2_ intercalation in the experimental spectrum of (IC_6_)_2_[PbI_4_] (Fig. 3A). Therefore, although the perovskite retains the 2D nature of its electronic structure near the band edges (Fig. 4B), I_2_ intercalation produces new electronic transitions making it a more isotropic light absorber.

#### Reduced dielectric confinement

We then computed the real component of the materials' frequency-dependent dielectric constant *ε*′ (Fig. S5†). Here also, only small changes occur in the plane parallel to the inorganic sheets (*ε*′_∥_) upon I_2_ intercalation whereas the dielectric response in the direction perpendicular to the inorganic sheets (*ε*′_⊥_) is strongly modulated. Therefore, not only is the *ε*′ of (IC_6_)_2_[PbI_4_]·2I_2_ increased compared to that of (IC_6_)_2_[PbI_4_] but it also shows significant anisotropy with a higher perpendicular component (*ε*′_⊥_) than a parallel component (*ε*′_∥_). This is also likely a result of the orientation of the I_2_ molecules, which provide polarizable electron density perpendicular to the inorganic sheets.

**Fig. 5:**
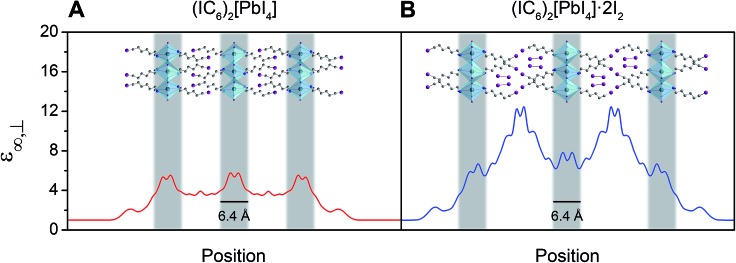
Slabs of (IC_6_)_2_[PbI_4_] (A) and (IC_6_)_2_[PbI_4_]·2I_2_ (B) and their corresponding calculated high-frequency dielectric profiles *ε*_∞,⊥_. Here, *ε*_∞,⊥_ is the high-frequency dielectric constant perpendicular to the direction of layer propagation. Dark green, purple, blue, and grey spheres represent Pb, I, N, and C atoms, respectively. Hydrogen atoms omitted for clarity.

The bulk high-frequency dielectric response (*ε*_∞_) corresponds to an average response over all layers of the perovskite and is not suited to describe the difference in dielectric constant between the organic and the inorganic layers. We therefore used a method designed to compute *ε*_∞_ profiles for nanoscale slabs and composite systems^10,51^ to estimate the respective contributions from the organic and inorganic layers to the bulk *ε*_∞_ value. Profiles of the high-frequency dielectric constant perpendicular to the layers (*ε*_∞,⊥_) in (IC_6_)_2_[PbI_4_] and (IC_6_)_2_[PbI_4_]·2I_2_ are shown in Fig. 5. The behavior of *ε*_∞,⊥_ changes substantially when I_2_ molecules intercalate. The average *ε*_∞,⊥_ dielectric profile stemming from the inorganic layers increases from 5.4 to 7.0 when including I_2_. For the *ε*_∞,⊥_ dielectric profile associated with the organic layers, we see a dramatic three-fold increase from 3.7 to 11.1 upon I_2_ intercalation. Therefore, I_2_ intercalation significantly decreases the dielectric confinement of excitons in the inorganic layers by better screening electric field lines in the organic layer.

Layered perovskites have been considered as quantum-well-like structures where the more polarizable (higher dielectric constant) inorganic sheets form the “wells” and the less polarizable organic layers (low dielectric constant) form the “barriers”.^8,38^ Notably, I_2_ intercalation completely inverts this dielectric profile to yield the first example of a 2D perovskite containing organic layers with a higher dielectric constant than the inorganic layers. The exciton's dielectric confinement is thus substantially reduced, though not completely eliminated because the exciton is likely screened only by the immediately adjacent portion of the organic layer.

#### Synergistic effects of intercalation

An accurate determination of *E*_b_ in a single quantum well with finite barriers or in a composite layered heterostructure can be calculated by including dielectric effects in the resolution of the Bethe–Salpeter equation (BSE) for the exciton to account for the abrupt interfaces between organic and inorganic components.^52,53^ Taking advantage of our *ab initio* determination of the perovskites' nanoscale dielectric profiles^10,51^ (Fig. 5), we refined this abrupt dielectric-interface approximation of the BSE for the first time to estimate the electron–hole coulombic interaction without experimental inputs, while simultaneously avoiding an unphysical divergence of the self-energy.^54^ This semi-empirical method (details in the ESI†) allows us to simulate the absorption spectra of large composite systems, in principle without experimental inputs, using the bandgaps and effective masses computed at the *ab initio* level. Here, we used the *E*_g_ values calculated at the HSE+SOC level of theory for (IC_6_)_2_[PbI_4_] and (IC_6_)_2_[PbI_4_]·2I_2_ including the self-energy corrections obtained from the computed dielectric profiles (Fig. S6; details in the ESI†). We also take into account the increase of the exciton's reduced mass upon I_2_ intercalation (from 0.11 to 0.13) predicted from the computed electronic band structures.

The resulting *E*_b_ values for (IC_6_)_2_[PbI_4_] and (IC_6_)_2_[PbI_4_]·2I_2_ are 288 and 171 meV, respectively. These numbers compare well with the experimental *E*_b_ values of 230 and 180 meV for (IC_6_)_2_[PbI_4_] and (IC_6_)_2_[PbI_4_]·2I_2_, respectively (determined using the exciton peak positions and bandgaps from the 5 K absorption spectra in Fig. 3B and C). Similar to our experimental absorption spectrum, the calculated spectrum for (IC_6_)_2_[PbI_4_]·2I_2_ (Fig. S7†) also shows a reduction in the ratio between the excitonic absorption peak and the above bandgap continuum compared to that of (IC_6_)_2_[PbI_4_], which is a good indicator of decreased dielectric confinement upon I_2_ intercalation (Fig. 3 and 5).^55^ The difference between the exciton peak positions in the computed absorption spectra for (IC_6_)_2_[PbI_4_] and (IC_6_)_2_[PbI_4_]·2I_2_ relative to experiment result from the difference between experimental and computed *E*_g_ and *E*_b_ values.

The experimentally observed reduction in the exciton's confinement upon I_2_ intercalation is therefore well supported by theory. Our calculations further indicate that the dominant contribution to this effect is the decrease in the exciton's dielectric confinement upon inclusion of the polarizable I_2_ molecules in the organic layers, which results in an inversion of the pseudo-quantum-well dielectric profile. The intercalating I_2_ molecules extend perpendicular to the inorganic sheets and further mitigate the 2D nature of the material's optical properties leading to more isotropic light absorption.

### 2.3. Dynamics of halogen intercalation

The foregoing calculations assumed that we experimentally accessed a single product through I_2_ intercalation: (IC_6_)_2_[PbI_4_]·2I_2_, with no intermediate species with varying I_2_ content. To test this assumption, we studied the dynamics of I_2_ intercalation. Because reaction with I_2_ vapor occurs in seconds, we designed *in situ* PXRD and electronic absorption spectroscopy cells for use with halogen gas to track potential reaction intermediates (see ESI for details and Fig. S8†).

**Fig. 6:**
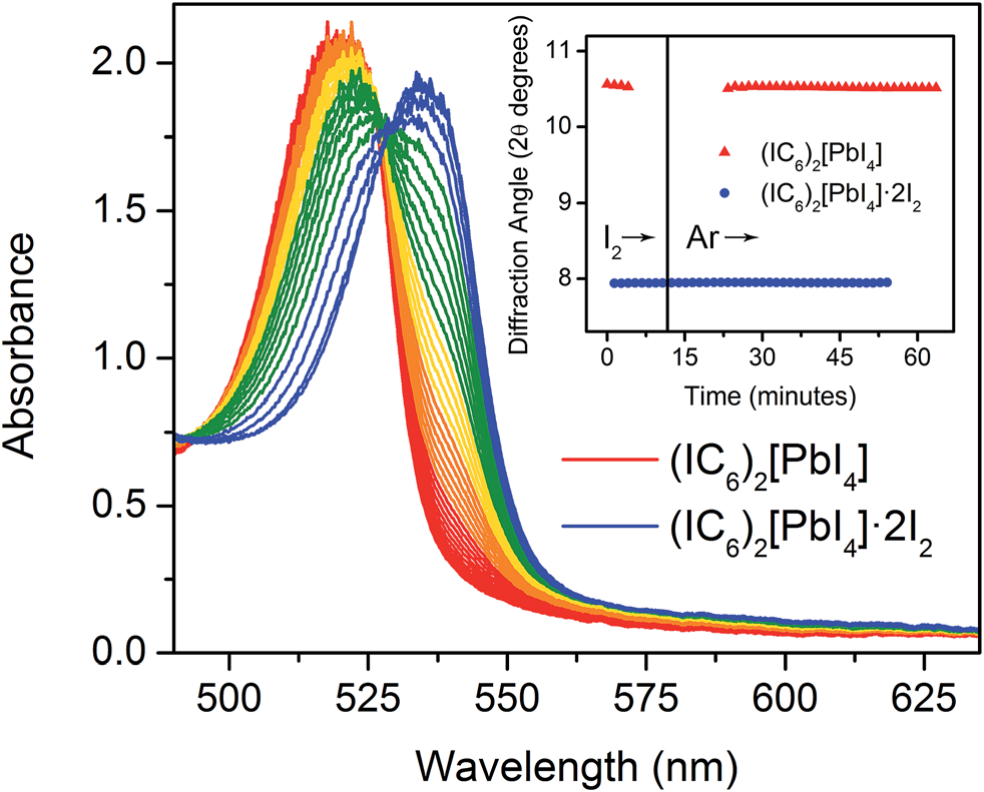
*In situ* optical absorbance spectra acquired as (IC_6_)_2_[PbI_4_]·2I_2_ (blue) desorbs I_2_ to yield (IC_6_)_2_[PbI_4_] (red). Inset: position of the (004) reflection in the powder X-ray diffraction pattern as (IC_6_)_2_[PbI_4_] absorbs and then desorbs I_2_ vapor.

We first studied the reaction between the alkyl perovskite (C_6_)_2_[PbI_4_] and I_2_. Here, we see evidence for reaction intermediates in both PXRD and optical absorption spectroscopy (see ESI† for details). The excitonic absorption band continuously redshifts upon I_2_ intercalation and it continuously blueshifts upon I_2_ release, indicating an evolving structure (Fig. S9 and S10†). In contrast, we did not observe any crystalline intermediates in the PXRD patterns during I_2_ absorption or desorption from the iodoalkyl perovskite (IC_6_)_2_[PbI_4_]·2I_2_ (Fig. 6, inset). The absence of intermediates is further supported by optical absorption spectroscopy. When we plot the exciton's spectral progression during I_2_ intercalation and deintercalation for (IC_6_)_2_[PbI_4_] and (IC_6_)_2_[PbI_4_]·2I_2_, respectively, we do not observe a continuous shift of the excitonic absorption energy (Fig. 6 and S11†). Instead, at intermediate scan times, the excitonic absorbance contains only contributions from the excitonic bands of (IC_6_)_2_[PbI_4_] and (IC_6_)_2_[PbI_4_]·2I_2_. The absence of intermediates in both the PXRD and absorption data suggests that I_2_ molecules that enter the perovskite rapidly localize to the binding sites provided by the organoiodines and inorganic iodides. Compared to (C_6_)_2_[PbI_4_], the organic layers in (IC_6_)_2_[PbI_4_] should interact more strongly with the inorganic sheets. Upon I_2_ loss from (IC_6_)_2_[PbI_4_]·2I_2_, the organic layers may more rapidly find their equilibrium positions in (IC_6_)_2_[PbI_4_] aided by iodine–iodine interactions between the organic and inorganic layers. Such halogen–halogen interactions have been previously invoked as structure-directing agents during the assembly of layered lead–halide perovskites.^41^ To further test if I_2_ molecules intercalate between the organic bilayers, we synthesized the alkyldiammonium perovskite (H_3_NC_6_H_12_NH_3_)[PbI_4_],^56^ which contains an organic monolayer that forms hydrogen bonds at both ends with adjacent inorganic sheets. We did not observe any new phases in the PXRD patterns during I_2_ exposure, indicating that an organic bilayer is necessary for I_2_ intercalation (Fig. S12†).

### 2.4. Halogen exchange

**Fig. 7:**
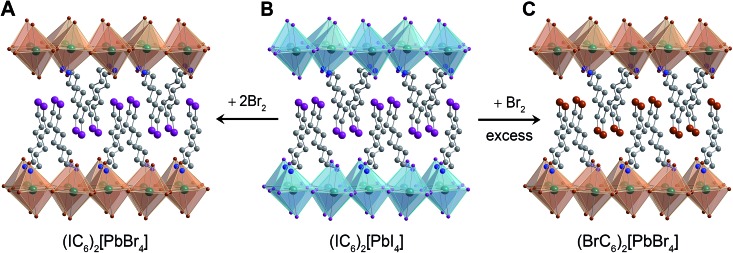
Schematic of products obtained through reaction of (IC_6_)_2_[PbI_4_] (B) with a stoichiometric amount of Br_2_ (A) and with excess Br_2_ (C). Turquoise and brown polyhedra represent Pb–I and Pb–Br octahedra, respectively. Dark green, purple, brown, blue, and grey spheres represent Pb, I, Br, N, and C atoms, respectively. Hydrogen atoms omitted for clarity.

We finally turned our attention to reactivity that may follow halogen intercalation. As we reported previously, Br_2_ exposure converts Pb–I perovskites to Pb–Br perovskites through redox-mediated halogen exchange.^25^ To further extend this reactivity, we investigated the reaction product of (IC_6_)_2_[PbI_4_] with Br_2_. In addition to inorganic halide substitution (95% conversion), analysis of the digested product by ^1^H NMR shows that the organoiodines have also been converted (90% conversion) to organobromines to yield the new perovskite: (BrC_6_H_12_NH_3_)_2_[PbBr_4_] (Fig. 7C, S13 and S17†). While atypical, a few examples of solution-state halogen-mediated organohalogen substitution reactions are known.^57,58^ To further parse the reactivity of the organic and inorganic components, we then studied the reaction between a 1 : 2 molar ratio of (IC_6_)_2_[PbI_4_] and Br_2_. The major product of this reaction (95% yield) was (IC_6_)_2_[PbBr_4_] (identified through ^1^H NMR, inductively coupled plasma mass spectrometry, and PXRD), where the organoiodines remain unsubstituted (Fig. 7A, S14 and S18†). This indicates that inorganic halide substitution precedes organohalogen exchange in the organic layer. Notably, we could not form this perovskite through solution-state reactions. When we combined IC_6_^+^ salts and PbBr_2_ in solution, Br^–^ ions partially displaced the organoiodines to form organobromines (Fig. S16†).

## 3. Conclusions

We demonstrate that small molecules can be stabilized in binding pockets designed into 2D perovskites. Notably, placing highly polarizable molecules in the organic layers can cause large changes in the electronic and optical properties of the inorganic sheets. Here we show that I_2_ intercalation results in more polarizable organic layers compared to the inorganic layers, which considerably decreases the dielectric confinement of excitons generated in the inorganic layers. Control over the exciton binding energy in 2D perovskites can enable their application in a broad range of optoelectronic technologies. Although the I_2_-intercalated perovskite studied here is metastable, our studies show that incorporation of polarizable functionalities through intercalation or covalent attachment in the organic layers is a viable approach for substantially decreasing the electronic confinement of these layered materials.

We further extend halogen intercalation to halogen-mediated reactivity where our studies show that inorganic halide exchange precedes organohalogen exchange. This gas–solid reaction allows us to synthesize perovskites that cannot be formed through traditional solution-state routes.

## References

[cit1] Mitzi D. B., in Prog. Inorg. Chem., ed. K. D. Karlin, John Wiley & Sons Inc, New York, 1999, vol. 48, pp. 1–121.

[cit2] Kojima A., Teshima K., Shirai Y., Miyasaka T. (2009). J. Am. Chem. Soc..

[cit3] Kazim S., Nazeeruddin M. K., Grätzel M., Ahmad S. (2014). Angew. Chem., Int. Ed..

[cit4] Snaith H. J. (2013). J. Phys. Chem. Lett..

[cit5] Lin Q. Q., Armin A., Nagiri R. C. R., Burn P. L., Meredith P. (2015). Nat. Photonics.

[cit6] Even J., Pedesseau L., Katan C. (2014). J. Phys. Chem. C.

[cit7] Muljarov E. A., Tikhodeev S. G., Gippius N. A., Ishihara T. (1995). Phys. Rev. B: Condens. Matter Mater. Phys..

[cit8] Even J., Pedesseau L., Katan C. (2014). ChemPhysChem.

[cit9] Keldysh L. V. (1979). Pis'ma Zh. Eksp. Teor. Fiz..

[cit10] Sapori D., Kepenekian M., Pedesseau L., Katan C., Even J. (2016). Nanoscale.

[cit11] Hanamura E., Nagaosa N., Kumagai M., Takagahara T. (1988). Mater. Sci. Eng., B.

[cit12] Even J., Pedesseau L., Dupertuis M.-A., Jancu J.-M., Katan C. (2012). Phys. Rev. B: Condens. Matter Mater. Phys..

[cit13] Takagi H., Kunugita H., Ema K. (2013). Phys. Rev. B: Condens. Matter Mater. Phys..

[cit14] Knupfer M. (2003). Appl. Phys. A: Mater. Sci. Process..

[cit15] Gauthron K., Lauret J.-S., Doyennette L., Lanty G., Al Choueiry A., Zhang S. J., Brehier A., Largeau L., Mauguin O., Bloch J., Deleporte E. (2010). Opt. Express.

[cit16] Era M., Morimoto S., Tsutsui T., Saito S. (1994). Appl. Phys. Lett..

[cit17] Dohner E. R., Hoke E. T., Karunadasa H. I. (2014). J. Am. Chem. Soc..

[cit18] Dohner E. R., Jaffe A., Bradshaw L. R., Karunadasa H. I. (2014). J. Am. Chem. Soc..

[cit19] Calabrese J., Jones N. L., Harlow R. L., Herron N., Thorn D. L., Wang Y. (1991). J. Am. Chem. Soc..

[cit20] Mitzi D. B., Wang S., Feild C. A., Chess C. A., Guloy A. M. (1995). Science.

[cit21] Hong X., Ishihara T., Nurmikko A. V. (1992). Phys. Rev. B: Condens. Matter Mater. Phys..

[cit22] Smith I. C., Hoke E. T., Solis-Ibarra D., McGehee M. D., Karunadasa H. I. (2014). Angew. Chem., Int. Ed..

[cit23] Slavney A. H., Smaha R. W., Smith I. C., Jaffe A., Umeyama D., Karunadasa H. I. (2016). Inorg.
Chem..

[cit24] Solis-Ibarra D., Karunadasa H. I. (2014). Angew. Chem., Int. Ed..

[cit25] Solis-Ibarra D., Smith I. C., Karunadasa H. I. (2015). Chem. Sci..

[cit26] Needham G. F., Willett R. D., Franzen H. F. (1984). J. Phys. Chem..

[cit27] Dolzhenko Y. I., Inabe T., Maruyama Y. (1986). Bull. Chem. Soc. Jpn..

[cit28] Jaffe A., Karunadasa H. I. (2014). Inorg. Chem..

[cit29] Mitzi D. B., Medeiros D. R., Malenfant P. R. L. (2002). Inorg. Chem..

[cit30] Billing D. G., Lemmerer A. (2007). Acta Crystallogr., Sect. B: Struct. Sci..

[cit31] Lemmerer A., Billing D. G. (2010). CrystEngComm.

[cit32] Politzer P., Murray J. S., Clark T. (2013). Phys. Chem. Chem. Phys..

[cit33] Mukherjee A., Tothadi S., Desiraju G. R. (2014). Acc. Chem. Res..

[cit34] Tasman H. A., Boswijk K. H. (1955). Acta Crystallogr..

[cit35] Castro-Castro L. M., Guloy A. M. (2003). Angew. Chem., Int. Ed..

[cit36] Daub M., Hillebrecht H. (2015). Angew. Chem., Int. Ed..

[cit37] Louvain N., Bi W., Mercier N., Buzaré J.-Y., Legein C., Corbel G. (2007). Dalton Trans..

[cit38] Ishihara T. (1994). J. Lumin..

[cit39] Hong X., Ishihara T., Nurmikko A. V. (1992). Solid State Commun..

[cit40] Knutson J. L., Martin J. D., Mitzi D. B. (2005). Inorg. Chem..

[cit41] Sourisseau S., Louvain N., Bi W., Mercier N., Rondeau D., Boucher F., Buzaré J.-Y., Legein C. (2007). Chem. Mater..

[cit42] Katan C., Pedesseau L., Kepenekian M., Rolland A., Even J. (2015). J. Mater. Chem. A.

[cit43] Mitzi D. B., Dimitrakopoulos C. D., Kosbar L. L. (2001). Chem. Mater..

[cit44] Shinada M., Sugano S. (1966). J. Phys. Soc. Jpn..

[cit45] Tanaka K., Takahashi T., Ban T., Kondo T., Uchida K., Miura N. (2003). Solid State Commun..

[cit46] Tanaka K., Takahashi T., Kondo T., Umeda K., Ema K., Umebayashi T., Asai K., Uchida K., Miura N. (2005). Jpn. J. Appl. Phys., Part 1.

[cit47] Heyd J., Scuseria G. E., Ernzerhof M. (2003). J. Chem. Phys..

[cit48] Heyd J., Scuseria G. E., Ernzerhof M. (2006). J. Chem. Phys..

[cit49] Paier J., Marsman M., Hummer K., Kresse G., Gerber I. C., Ángyán J. G. (2006). J. Chem. Phys..

[cit50] Umebayashi T., Asai K., Kondo T., Nakao A. (2003). Phys. Rev. B: Condens. Matter Mater. Phys..

[cit51] Even J., Pedesseau L., Kepenekian M. (2014). Phys. Chem. Chem. Phys..

[cit52] Guseinov R. R. (1984). Phys. Status Solidi B.

[cit53] Thoai D. B. T., Zimmermann R., Grundmann M., Bimberg D. (1990). Phys. Rev. B: Condens. Matter Mater. Phys..

[cit54] Even J., Pedesseau L., Jancu J.-M., Katan C. (2013). J. Phys. Chem. Lett..

[cit55] Rodina A. V., Efros A. L. (2016). J. Exp. Theor. Phys..

[cit56] Mousdis G. A., Papavassiliou G. C., Raptopoulou C. P., Terzis A. (2000). J. Mater. Chem..

[cit57] Friedel C. (1865). Justus Liebigs Ann. Chem..

[cit58] Corey E. J., Wechter W. J. (1954). J. Am. Chem. Soc..

