# Supplementary material for: Decreasing the electronic confinement in layered perovskites through intercalation
Source: Chem Sci. 2016 Nov 10;8(3):1960–8. doi: 10.1039/c6sc02848a (PMC5384564; doi:10.1039/c6sc02848a)
Supplement: Supplementary file 1 [file SC-008-C6SC02848A-s001.pdf]

Electronic Supplementary Material (ESI) for Chemical Science.
This journal is © The Royal Society of Chemistry 2016

Supplementary Information for:

Decreasing the Electronic Confinement in Layered Perovskites through Intercalation

Matthew D. Smith,[a] Laurent Pedesseau,[b] Mikaël Kepenekian,[c] Ian C. Smith,[a] Claudine Katan,[c] Jacky Even,[b,*] and Hemamala I. Karunadasa[a,*]

[a] *Department of Chemistry, Stanford University, Stanford, CA 94305, United States*

[b] *Fonctions Optiques pour les Technologies de l'information, CNRS, INSA de Rennes, 35708 Rennes, France*

[c] *Institut des sciences chimiques de Rennes, CNRS, Université de Rennes 1, 35042 Rennes, France*

[*] hemamala@stanford.edu, Jacky.Even@insa-rennes.fr

Experimental Methods

Tables S1–S3

Figures S1–S22

References

Experimental Methods

All manipulations were conducted in air unless otherwise stated. Organic solvents were of reagent grade or higher purity. The solvents *N*,*N*′-dimethylformamide (DMF) and benzene were dried and degassed using the JC Meyer solvent purification system. All other reagents were purchased from commercial vendors and used as received. Concentrated hydroiodic acid (55-57% w/w containing 1.5% hypophosphorous acid as stabilizer) was used as received. Inductively coupled plasma mass spectrometry (ICP-MS) blank and standard solutions, all 5% aqueous HNO_3 solutions, were purchased from Inorganic Ventures and used as received.

Hexane-1,6-diaminium iodide, $(H_3NC_6H_{12}NH_3)I_2$

The ammonium salt was prepared by dissolving 0.332 g (2.86 mmol) of 1,6-diaminohexane in 2.0 mL of concentrated hydroiodic acid. The solution was heated to 50 °C and held under reduced pressure until a colorless solid was recovered. The solid was filtered, washed with diethyl ether (10 mL × 2), and held at reduced pressure to yield 0.909 g of colorless powder (yield 85.5%). ^{1}H NMR (DMSO-d_6): 7.62 (br, 6H); 2.81-2.73 (m, 4H); 1.53-1.45 (m, 4H); 1.33-1.23 (m, 4H).

6-iodohexan-1-aminium iodide, $(IC_6H_{12}NH_3)I$

The ammonium salt was prepared according to a modified literature procedure.[1] In a round-bottom flask, 0.605 g (5.16 mmol) of 6-aminohexan-1-ol was dissolved in 5 mL of concentrated hydroiodic acid. The flask headspace was sparged with N_2 and the solution was heated at 80 °C for 23 h. The solvent was removed using reduced pressure at 50 °C to recover a light yellow solid. This solid was filtered, washed with diethyl ether (10 mL × 2), and held at reduced pressure to yield 1.06 g of colorless powder (yield 57.7%). ^{1}H NMR (DMSO-d_6): 7.60 (br, 3H); 3.28 (t, 2H); 2.81-2.73 (m, 2H); 1.79-1.72 (m, 2H); 1.55-1.48 (m, 2H); 1.39-1.27 (m, 4H).

6-iodohexan-1-aminium tosylate, $(IC_6H_{12}NH_3)(CH_3C_6H_4SO_3)$

A 1-mL methanol solution of 6-iodohexan-1-aminium iodide (1.79 g, 0.503 mmol) at 0 °C was added dropwise to a 60-mL methanol solution of silver(I) tosylate (0.140 g, 0.502 mmol) and the mixture was allowed to stir in the dark for an additional 10 minutes at 0 °C. The resulting colorless suspension was filtered through glass filter paper to recover a clear solution. The solvent was removed from the filtrate at reduced pressure to afford 0.176 g (87.8%) of 6-iodohexan-1-aminium tosylate. ^{1}H NMR (DMSO-d_6): 7.59 (br, 3H); 7.47 (m, 2H); 7.11 (m, 2H); 3.28 (t, 2H); 2.76 (m, 2H); 2.29 (s, 3H); 1.78-1.71 (m, 2H); 1.55-1.47 (m, 2H); 1.39-1.29 (m, 4H).

$(C_6H_{13}NH_3)_2[PbI_4]$ perovskite, $(C_6)_2[PbI_4]$

Powder of $(C_6)_2[PbI_4]$ was prepared according to a modified literature procedure.[2] Solid PbI_2 (0.369 g, 0.800 mmol) was dissolved in 2 mL of concentrated hydroiodic acid and sonicated for 5 minutes. Then 1-hexylamine (0.220 mL, 1.67 mmol) was added to this solution. The resulting red-orange suspension was heated at 100 °C for 3 h and then cooled to room temperature. The resulting red-orange crystals were filtered through a glass frit and washed with diethyl ether. The red-orange solid was then ground and held at reduced pressure to afford 0.531 g (72.1% yield) of product. The recovered powder was stored in a desiccator in the dark to inhibit decomposition.

$(IC_6H_{12}NH_3)_2[PbI_4]$ perovskite, $(IC_6)_2[PbI_4]$

Powder of $(IC_6)_2[PbI_4]$ was prepared according to a modified literature procedure.[2] Solid PbI_2 (0.455 g, 0.987 mmol) and 6-amino-1-hexanol (0.272 g, 2.32 mmol) were dissolved in 2 mL of concentrated hydroiodic acid and sonicated for 5 minutes. The resulting yellow-orange solution was heated at 100 °C for

3 h and then cooled in air to room temperature. The resulting red-orange crystals were filtered through a glass frit and washed with diethyl ether. The red-orange solid was then ground and held at reduced pressure to afford 1.03 g (88.8% yield) of product. The recovered powder was stored in a dessicator in the dark to inhibit decomposition.

Preparation of oriented films

Glass or quartz slides were sequentially cleaned via sonication in aqueous Sparkleen® detergent, deionized water, acetone, and isopropanol. The slides were finally treated with a 15-minute UV-ozone etch immediately prior to use. Oriented films of the lead-iodide perovskites (with the inorganic sheets parallel to the substrate) were deposited by spincoating. Typically, a 0.4-M DMF solution of the lead-iodide perovskite was sonicated and then filtered through glass filter paper or 0.22-μm pore size Teflon syringe filters. Then 50-100 μL of the solution was deposited on the slide, which was then spun at 3000 rpm for 50 s and then at 5000 rpm for 10 s. The films were annealed at 100 °C for 10 minutes before cooling to room temperature in air.

Halogen conversion reactions

We established the composition of the brominated perovskites $(BrC_6H_{12}NH_3)_2[PbBr_4]$ and $(IC_6H_{12}NH_3)_2[PbBr_4]$ through a combination of PXRD, ^{1}H NMR, and ICP-MS. Following exposure of $(IC_6)_2[PbI_4]$ to Br_2, we first verified that the PXRD patterns of the recovered products did not match those of the precursors (Figures S13-S15). Preliminary analysis suggests that the interlayer spacing in the Pb–Br lattice is greater than that of the Pb–I lattice, which may be due to differing degrees of interdigitation in the organic bilayer. We have previously observed a contraction of the interlayer spacing when moving to perovskites with larger halides.[3] Based on single-crystal X-ray analysis, the distance between inorganic sheets in $(HBrCCHBr(CH_2)_2NH_3)_2[PbCl_4]$ is larger than this distance in $(HBrCCHBr(CH_2)_2NH_3)_2[PbBr_4]$. Within the inorganic layers, the next-nearest neighbor Pb···Pb distances are larger in the Pb–Br lattice, as expected. Therefore, upon substitution of Pb–Br for Pb–Cl, the intralayer Pb···Pb distance decreases, yet the interlayer distance increases. Such non-systematic variation in lattice parameters is common in these materials.

We then calculated the total Pb:Br:I ratio through ICP-MS data. Using the ratio of Br:I in the organic layer, as determined by ^{1}H NMR of the digested samples (Figures S17 and S18), we then determined the Br:I ratio in the inorganic layer.

We also tested if the organic cations alone would react similarly with bromine gas. Our control reactions showed that Br_2 vapor can replace the iodine atoms in solid samples of both the iodide and tosylate salts of IC_6. We obtained very low yields (6–7% yields) in these control reactions, likely because in contrast to the perovskites, which allows for intercalation throughout the material, only the exterior regions of the solid IC_6^+ salts reacted with Br_2 gas (Figures S21 and S22).

Perovskite bromination reactions

To preclude the formation of halogen radicals, all manipulations were conducted in the dark unless stated otherwise. Bulk powder samples of $(IC_6)_2[PbI_4]$ were suspended in anhydrous 1,2-dichlorobenzene and milled for 8 h at 800 cycles·min^{-1} on a Fritsch Pulverisette 7 planetary ball mill. This powder was then isolated and dried under reduced pressure and dispersed in a solution of bromine in 80 mL of anhydrous benzene, sealed, covered with foil, and allowed to stir for 5 days at room temperature. The precipitate was recovered using nylon filter paper and washed with a copious amount of anhydrous benzene. For partial bromination, the molar ratio of Br_2:$(IC_6)_2[PbI_4]$ was 2:1. For complete bromination, the molar ratio of

Br_2:$(IC_6)_2[PbI_4]$ was 6:1. The recovered colorless solid was held at reduced pressure to remove any remaining volatiles. The samples were analyzed by PXRD, ^{1}H NMR, and ICP-MS.

Bromination of 6-iodohexan-1-aminium salts

To prevent the formation of halogen radicals, all manipulations were conducted in the dark unless stated otherwise. Solid 6-iodohexan-1-aminium iodide (0.034 g, 0.096 mmol) that was freshly washed with diethyl ether was added to a small paper funnel and held at reduced pressure to remove moisture and iodine. The sample was then placed on a glass frit connected to a custom-made bromine vapor flow system. In the system, N_2 was flowed over a round-bottom flask filled with bromine at a rate of 40 mL·min^{-1}. This flow was diluted with an additional N_2 flow of 2 L·min^{-1} prior to reaching the sample. The sample was exposed to this diluted gas mixture for 10 minutes. The resulting 0.030 g of orange gel was held at reduced pressure to remove volatile side products and was analyzed by ^{1}H NMR.

Solid 6-iodohexan-1-aminium tosylate (0.016 g, 0.040 mmol) contained in a small paper funnel was placed on a glass frit connected to a bromine flow system. Then N_2 was flowed over a round-bottom flask filled with bromine at a rate of 40 mL·min^{-1} for 10 minutes. This flow was diluted with an additional N_2 flow of 2 L·min^{-1} for 10 minutes. The resulting 0.011 g of yellow solid was held at reduced pressure to remove volatile side products and was analyzed by ^{1}H NMR.

Powder X-ray diffraction (PXRD)

Experiments on powders and oriented films were conducted on a Panalytical X'Pert Pro diffractometer with a Bragg-Brentano geometry with programmable divergence slits equipped with a nickel filter and PIXCEL 1D detector with copper anode ($K\alpha_1$ = 1.54060 Å, $K\alpha_2$ = 1.54443 Å, $K\alpha_2/K\alpha_1$ = 0.50000, step size 0.02 °2θ). In situ PXRD measurements with I_2 used a custom gas-tight flow stage (Figure S8).

Crystal structure determination

A crystal was coated with Paratone-N oil, attached to a Mitegen loop or micromesh mount, and transferred to a Bruker D8 Venture diffractometer equipped with a Photon 100 CMOS detector. Frames were collected using ω and ϕ and scans and the unit-cell parameters were refined against all data. Data were integrated and corrected for Lorentz and polarization effects using SAINT v8.34A and were corrected for absorption effects using SADABS V2014/2.[4] The structure was solved using the intrinsic phasing method implemented in APEX2. It was refined against all data using SHELXTL[5, 6] and OLEX2[7] software.[8] Hydrogen atoms were inserted at idealized positions and refined using a riding model with an isotropic thermal parameter 1.2 or 1.5 times that of the attached carbon or nitrogen atom, respectively. Thermal parameters for all non-hydrogen atoms were refined anisotropically.

In situ optical measurements

In situ optical absorbance spectra were acquired using an OceanOptics USB4000 spectrometer with a mercury lamp light source or a Cary 6000i spectrometer. For time-dependent measurements, spectra were acquired over 100 ms and then 10 scans were averaged. Films on glass were held in a quartz cuvette with a custom 3D-printed poly(lactic acid) cap to allow for nitrogen and iodine flow over the sample (Figure S8).

Inductively coupled plasma mass spectrometry (ICP-MS)

Data were collected at the Stanford University Environmental Measurements Facility. Typically, 10 mg of a perovskite was dissolved in 5 mL of 5% aqueous HNO_3 solution, sonicated, and filtered through 0.22-μm

poly(vinylidene fluoride) (PVDF) membranes. The resulting solution was diluted 100-fold and used for ICP-MS analysis for lead (Pb) and bromine (Br) content.

Other physical measurements

Room-temperature optical absorbance spectra for thin films were acquired on an Agilent Cary 6000i spectrometer. Low-temperature absorbance spectra were acquired on a Cary 500 spectrometer. Films were deposited on quartz discs and cooled to 5 K using a Janus STVP-100 optical continuous flow cryostat. The thickness of the films was determined using a Dektak 3 Profilometer. NMR spectra were recorded on a Varian Mercury 400 MHz instrument and peaks were referenced to residual solvent peaks. Samples were dissolved in DMSO-d_6 (δ 2.50) or methanol-d_4 (δ 3.31). Data are reported as follows: chemical shift (multiplicity, integration).

Computational details

Different theoretical and computational approaches are implemented in this work. For structural optimization and electronic structure calculation, density functional theory (DFT) calculations are performed using the plane-wave projector augmented wave (PAW) method[9, 10] as implemented in the VASP code[11-13]. We used the PAW data set supplied in the VASP-PAW package with the following valence orbitals: Pb [$5d^{10}6s^26p^2$], I [$5s^25p^5$], N [$2s^22p^3$], H [$1s^1$] and C [$2s^22p^2$] atoms. In addition, the wavefunctions are expanded using a plane-wave basis set with an energy cut-off of 500 eV.

For structural optimizations, both internal coordinates and cell parameters are relaxed. After a first relaxation using the local density approximation (LDA), the final structure is determined using the generalized gradient approximation (GGA) optimized to take into account weak interactions as proposed by Dion and co-workers and revised by Klimeš et al. The functional used for these calculations is the optB88-vdW functional (hereafter denoted as GGA+vdW).[14-16] The structures are relaxed until the forces are smaller than 10^{-4} eV/Å. Except for some values reported in Tables S2 and S3, all physical properties are computed using the perovskite structures that have been optimized at the GGA+vdW level of theory.

Subsequent calculations to determine projected density of states (pDOS) and electronic band structures are performed using three different levels of theory (i) GGA+vdW, (ii) GGA+vdW with the addition of spin-orbit coupling (SOC; hereafter denoted as GGA+vdW+SOC) and (iii) the Heyd, Scuseria and Ernzerhof (HSE) hybrid functional with SOC (hereafter denoted as HSE+SOC).[17-19] Except for the well-known variation of the bandgap, we checked that no qualitative difference arises between the HSE+SOC and GGA+vdW+SOC levels of theory (Figure S3).

In the case of band structure calculations, the reciprocal space integration is performed over a 4×4×1 Monkhorst-Pack grid.[20, 21] The pDOS and dielectric responses are computed using a 8×8×1 grid Monkhorst-Pack grid. As it is computationally unaffordable, it has not been possible to compute dielectric responses at the HSE+SOC level. We therefore used the GGA+vdW+SOC level of theory for these calculations.

Dielectric profiles are calculated using the SIESTA package.[22, 23] Core electrons are described with Troullier-Martins pseudopotentials[24], while the valence wavefunction is developed over a double-zeta polarized basis set of finite-range numerical pseudoatomic orbitals.[25] We use the aforementioned optB88-vdW functional together with on-site corrections for SOC.[26] A more detailed description of the computation of dielectric and self-energy profiles can be found in our previous work.[27, 28]

Rydberg model for the exciton binding energy (E_b)

The E_b in 2D perovskites is much higher than the E_b of 3D perovskites owing to quantum confinement and dielectric confinement. To estimate the magnitude of these two contributions to the E_b of the 2D perovskite, we calculated the E_b for the 2D perovskite considering only quantum confinement effects. For this, we first computed the E_b of the material assuming it was electronically 3D by using the hydrogenic Bohr model

modified by the maximum dielectric constant of the inorganic layers (ϵ_{well}) and the computed electron-hole reduced mass (μ) for the 2D perovskite. This gives the pseudo-Rydberg energy or $E_{b,3D}$:

$$E_{b,3D} = Ry = \frac{\mu e^4}{8\epsilon_{well}^2 h^3 c} \quad (1)$$

Here, $E_{b,3D}$ is the E_b of the electronically 3D material, h is Planck's constant, c is the speed of light, and e is the charge of an electron. With respect to the hydrogenic Bohr model, the vacuum dielectric constant has been replaced with the maximum dielectric constant of the inorganic well layers (5.4 and 7.0, without or with I_2, respectively), and the free electron mass has been replaced by the electron-hole reduced mass. The E_b of the electronically 2D material (considering only quantum confinement) can then be estimated as $E_{b,2D} = 4E_{b,3D}$.[29, 30] Our experimental E_b values are higher that these computed values, indicating the contribution of the exciton's dielectric confinement to its E_b.

Self-energy corrections to the bandgap and exciton binding energy

The various contributions to the electronic bandgap of 2D/3D structures can be estimated from theoretical calculations by using the following decomposition:[27, 28]

$$E_g \approx E_{g;DFT,bulk} + \Sigma_{bulk} + \delta E_{g,DFT} + \delta\Sigma, \quad (2)$$

where $E_{g;bulk} = E_{g;DFT,bulk} + \Sigma_{bulk}$ is the bulk bandgap evaluated either from experiment or at the DFT level, including self-energy corrections due to many-body effects. Effects of quantum confinement and lattice distortions can be estimated from $\delta E_{g,DFT}$. For pure 2D hybrid perovskites with similar effective masses, variations of $\delta E_{g,DFT}$ are essentially expected from lattice distortions. In the present work, the self-energy variations $\delta\Sigma$ are mainly associated to chemically induced changes of the dielectric properties of the layered heterostructures.

Starting from a classical modelling of the dielectric profile at the nanoscale, $\delta\Sigma$ can be estimated for the monoelectronic states close to the bandgap. Until recently, analyses of the effect of dielectric confinement on the exciton resonances relied on a crude modeling of the dielectric profile through the heterostructure, namely an ad hoc abrupt dielectric interface separating layers with bulk-like dielectric constants.[31] This approach has several severe limitations. It leads to unphysical mathematical divergences of the self-energy profile at the interfaces.[27, 28] It is also difficult to exactly define the size of the layers, as an atomistic description of the interfaces is lacking. Finally, the dielectric constants of the inorganic layers are approximated as the bulk dielectric constant of the corresponding 3D perovskite $(CH_3NH_3)PbX_3$ (X = Br, Cl, I)[32] or lead halide[33], even for the case of the very thin (n = 1) inorganic layers.

We have recently shown that DFT can be used to calculate dielectric profiles of layered systems with improved accuracy, yet still at a reasonable computational cost.[27, 28] This approach takes advantage of the ab initio description of nanoplatelets or 2D/3D heterostructures, and in particular of interfaces. The planar average of the self-energy correction $\delta\Sigma(z)$ can be derived from the transverse Fourier transform of the electrostatic potential $V(q, z, z_0)$ produced at position z by a charge e_0 located at position z_0:

$$\delta\Sigma(z_0) = \frac{e_0}{4\pi}\int_0^\infty \left(V(q, z, z_0) - V_{bulk}(q, z, z_0)\right)_{z\to z_0} q\,dq. \quad (3)$$

$V(q, z, z_0)$ is obtained by solving the inhomogeneous Poisson equation:

$$\frac{\partial}{\partial z}\left(\varepsilon(z)\frac{\partial}{\partial z}(V(q, z, z_0))\right) - q^2\varepsilon(z)V(q, z, z_0) = -e_0\delta(z - z_0). \quad (4)$$

For a homogeneous bulk material, the analytical solution reads:

$$V_{bulk}(q, z, z_0) = \frac{e^{-q|z-z_0|}}{2q\varepsilon} \quad (5)$$

Equation (5) shows that the dielectric profile $\varepsilon(z)$, i.e. along the principal axis ($\varepsilon_{\infty,\perp}(z)$) of the slab of 2D perovskite (Figure 5 and S6), is needed to accurately compute $V(q,z,z_0)$ and subsequently $\delta\Sigma(\mathrm{z})$ according to equation (3).

Figures S6E and S6F display the calculated self-energy corrections implementing this approach for slabs of $(IC_6)_2[PbI_4]$ and $(IC_6)_2[PbI_4]\cdot 2I_2$, respectively. Each slab is composed of three adjacent inorganic layers and their respective hydrogen-bonded ammonium cations. At the edges of both slabs, the last peak and minima in the self-energy correction are a result of the organic molecules that passivate the outermost inorganic layers. One notable feature is that while the organic component between the inorganic layers in $(IC_6)_2[PbI_4]$ undergoes a tiny correction of the self-energy of ca. +0.01 eV, the I_2-intercalated slab exhibits a correction of ca. +0.10 eV in the same spatial region. As a result, the fluctuations in self-energy between the organic and inorganic layers are quite small in the middle of the $(IC_6)_2[PbI_4]\cdot 2I_2$ slab.

Next, we obtain the semi-classical evaluation of $\delta\Sigma$ for both the conduction band (CB) and the valence band (VB) states by computing the following integrals over a unit cell, taking the central part of $\delta\Sigma(\mathrm{z})$ from Figures S6E and S6F:

$$\delta\Sigma_{CB(VB)} \approx \int \delta\Sigma(z)\rho_{CB(VB)}(z)dz \qquad (6)$$

$\rho_{CB(VB)}(z)$ is the electronic density profile for the CB (VB) state. The self-energy corrections are then estimated to be $\delta\Sigma_{CB}$ = 90 meV ($\delta\Sigma_{VB}$ = 65 meV) for the $(IC_6)_2[PbI_4]$ slab and $\delta\Sigma_{CB}$ = 18 meV ($\delta\Sigma_{VB}$ = 13 meV) for the $(IC_6)_2[PbI_4]\cdot 2I_2$ slab. The bandgap correction $\delta\Sigma = \delta\Sigma_{CB} + \delta\Sigma_{VB}$ is therefore reduced for $(IC_6)_2[PbI_4]\cdot 2I_2$ (by 31 meV) as compared to $(IC_6)_2[PbI_4]$ (by 155 meV). Taking these self-energy corrections into account, the corrected band gap of bulk $(IC_6)_2[PbI_4]\cdot 2I_2$ is 230 meV lower than the corrected band gap of bulk $(IC_6)_2[PbI_4]$ (Figure S7).

Finally, to compute the full absorption spectrum including excitonic effects, we still need to include the electron-hole interaction. Details will be published in a forthcoming publication. In short, this is implemented semi-empirically by solving the Bethe-Salpeter equation (BSE) based on the work by Chuang et al.[34], considering $z = z_0 = 0$ due to the 2D character of the material of interest. The exciton Green's function is solved according to equation (19) of reference 34. The optical susceptibility is computed using equation (14) of reference 34. The effective mass approximation is used for the energies (equation (11) of reference 34), with effective masses extracted from the HSE+SOC VB and CB dispersions. Corresponding wavefunctions (equations (8a) and (8b) of reference 34) are also deduced from the DFT computed electron densities at the center of the Brillouin zone.

Then, considering the corrected bandgaps determined in the previous paragraph, we obtain the absorption spectra, including excitonic effects (Figure S7). According to this procedure, the exciton's binding energy amounts to 288 and 171 meV, for $(IC_6)_2[PbI_4]$ and $(IC_6)_2[PbI_4]\cdot 2I_2$, respectively. These computed values qualitatively agree with the experimentally observed decrease of the exciton's binding energy upon I_2 intercalation.

Table S1. Crystallographic data[a] for $(IC_6)_2[PbI_4]$

	$(IC_6)_2[PbI_4]$
Empirical Formula	$C_{12}H_{30}N_2PbI_6$
Formula Weight, g·mol^{-1}	1170.97
Temperature, K	298(2)
Crystal System	Orthorhombic
Space group	*Pbca*
a, Å	9.0651(11)
b, Å	8.8852(10)
c, Å	33.617(5)
Volume, Å^3	2707.7(6)
Z	4
Density (calculated), g·cm^{-3}	2.872
Absorption coefficient, mm^{-1}	13.070
F(000)	2064.0
Crystal size, mm^3	0.2 × 0.1 × 0.05
θ range, °	2.553 to 25.358
Index ranges	$-10 \leq h \leq 10$<br>$-10 \leq k \leq 9$<br>$-40 \leq l \leq 40$
Reflections collected/unique	48334/2479
Completeness to θ_{max}	0.999
Max. and min. transmission	0.332, 0.241
Data/restraints/parameters	2479/191/152
Goodness-of-fit on F^2	1.123
Final *R* indices $[I > 2\sigma(I)]$[b]	$R_1 = 0.0315$<br>$wR_2 = 0.0661$
R indices (all data)[b]	$R_1 = 0.0430$<br>$wR_2 = 0.0706$
Largest diff. peak and hole, e·Å^{-3}	0.922, –0.791

[a]Obtained with monochromated Mo Kα (λ = 0.71073 Å) radiation
[b]$R_1 = \Sigma||F_o| - |F_c||/\Sigma|F_o|$, $wR_2 = [\Sigma w(F_o^2 - F_c^2)^2/\Sigma(F_o^2)^2]^{1/2}$

Table S2. Selected structural data of the $(IC_6)_2[PbI_4]$ experimental and optimized crystal structures, and of the $(IC_6)_2[PbI_4]\cdot 2I_2$ optimized structure. Unit-cell parameters (a, b, c (Å)), selected atomic bond lengths (Å) and octahedral tilt angles (β, δ (°)). The angle β describes in-plane octahedral torsion, while δ measures the octahedral tilt angle (Figure S1).[35]

	$(IC_6)_2[PbI_4]$				$(IC_6)_2[PbI_4]\cdot 2I_2$		
	298 K	173 K[2]	LDA (DFT)	GGA+ vdW (DFT)	298 K	LDA (DFT)	GGA+ vdW (DFT)
a, Å	9.065	9.221	8.83	8.93	-	8.69	8.66
b, Å	8.885	8.851	8.42	8.56	-	8.34	8.32
c, Å	33.617	31.980	31.92	32.50	44.511	44.41	44.95
$C_6I\cdots IC_6$, Å	5.017	4.988	5.12	5.15	-	5.20	5.16
$C_6I\cdots I$–Pb, Å	3.955	3.876	3.54	3.62	-	-	-
Pb–I *terminal*, Å	3.188	3.172	3.15	3.19	-	3.19	3.20
Pb–I *bridging*, Å	3.215	3.232	3.14	3.18	-	3.16	3.13
	3.231	3.248	3.15	3.18	-	3.17	3.14
I–I (iodine), Å	-	-	-	-	-	2.82	2.79
$C_6I\cdots I_2$, Å	-	-	-	-	-	3.22	3.32
$I_2\cdots I$–Pb, Å	-	-	-	-	-	3.27	3.33
β, °	10.1	9.5	14.2	13.6		18.0	16.8
δ, °	8.2	9.8	6.1	5.8		7.5	5.9

Table S3. Bandgaps (E_g (eV)) computed at various levels of theory for $(IC_6)_2[PbI_4]$ and $(IC_6)_2[PbI_4]\cdot 2I_2$ structures optimized at the LDA and GGA+vdW levels.

Compound	$(IC_6)_2[PbI_4]$				$(IC_6)_2[PbI_4]\cdot 2I_2$			
Structure optimization level of theory	LDA		GGA+vdW		LDA		GGA+vdW	
Electronic property calculation level of theory	LDA+ SOC	HSE+ SOC	GGA+ vdW+ SOC	HSE+ SOC	LDA+ SOC	HSE+ SOC	GGA+ vdW+ SOC	HSE+ SOC
E_g, eV	1.12	2.02	1.31	2.15	0.96	1.86	1.15	2.04

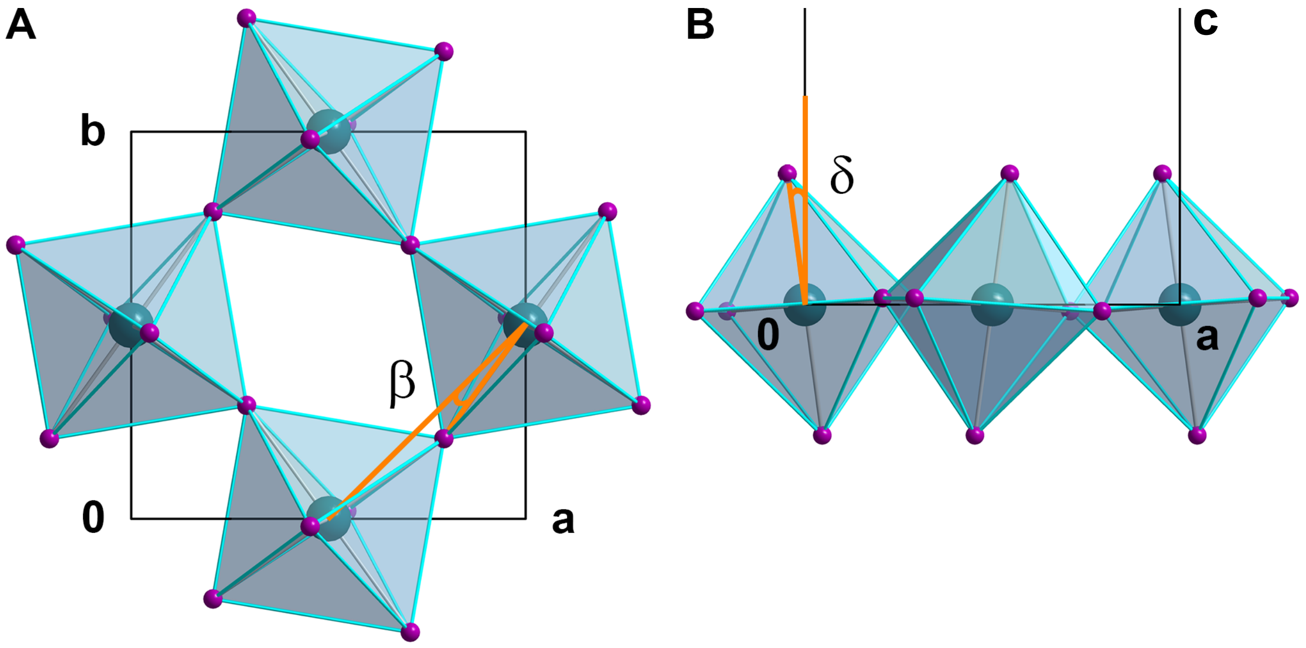

Figure S1. Schematic of the angle *β*, which describes in-plane octahedral torsion (A). Schematic of the angle *δ*, which describes the octahedral tilt angle (B). Dark green and purple spheres represent Pb and I atoms, respectively.

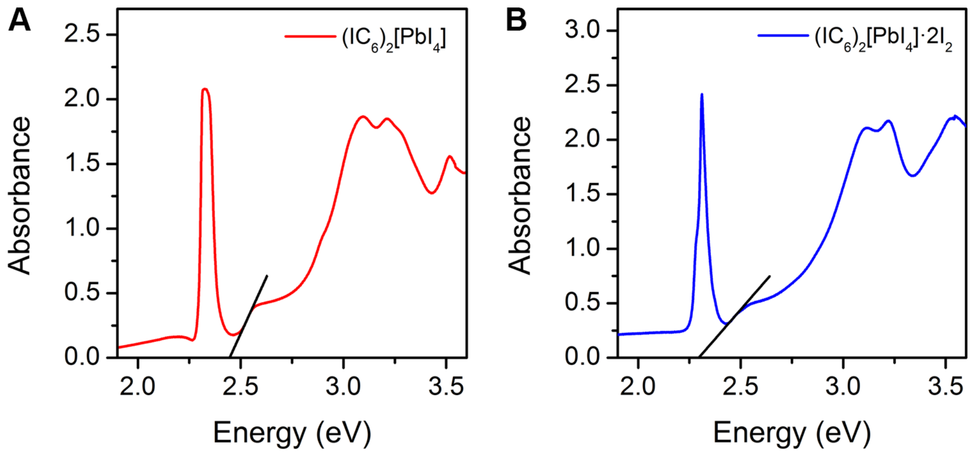

Figure S2. Close-up of the excitonic absorption and bandgap absorption in $(IC_6)_2[PbI_4]$ (A) and $(IC_6)_2[PbI_4]\cdot 2I_2$ (B). The black lines were used to estimate the bandgap in each material.

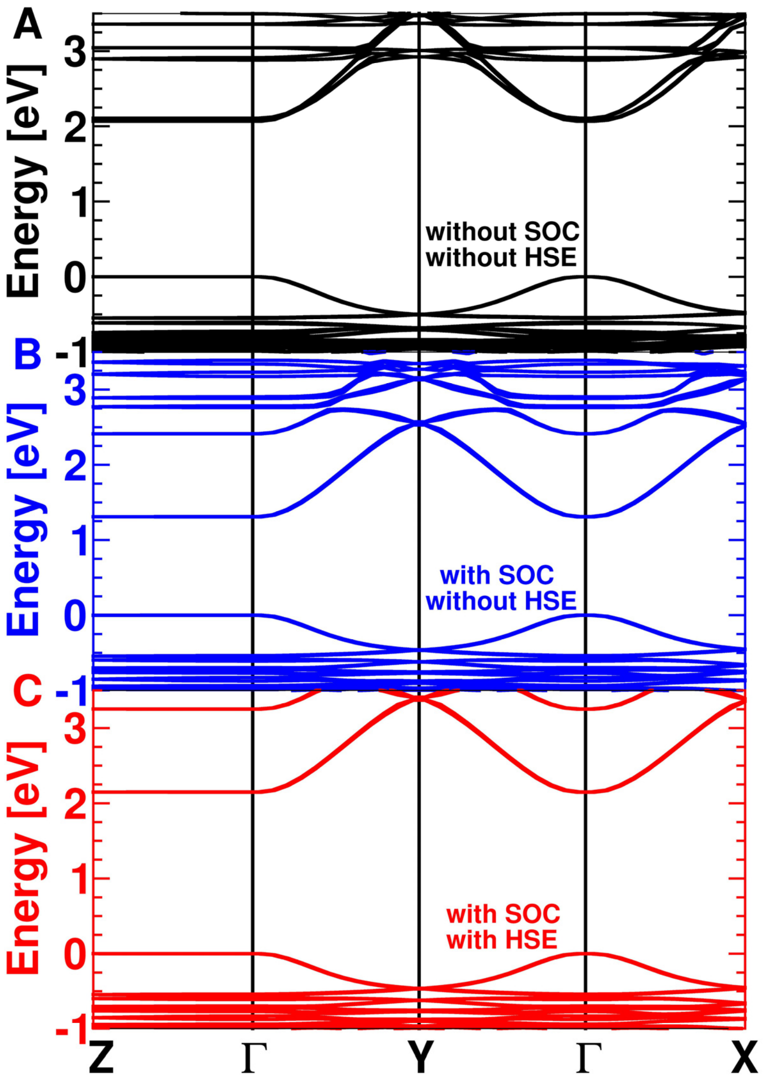

Figure S3. DFT electronic band structures of $(IC_6)_2[PbI_4]$ computed at the GGA+vdW level of theory (A, black), with the addition of SOC (B, blue) and using the HSE (μ = 0.1) hybrid functional (C, red) and including SOC effects. The energy of the valence band maximum is set at zero.

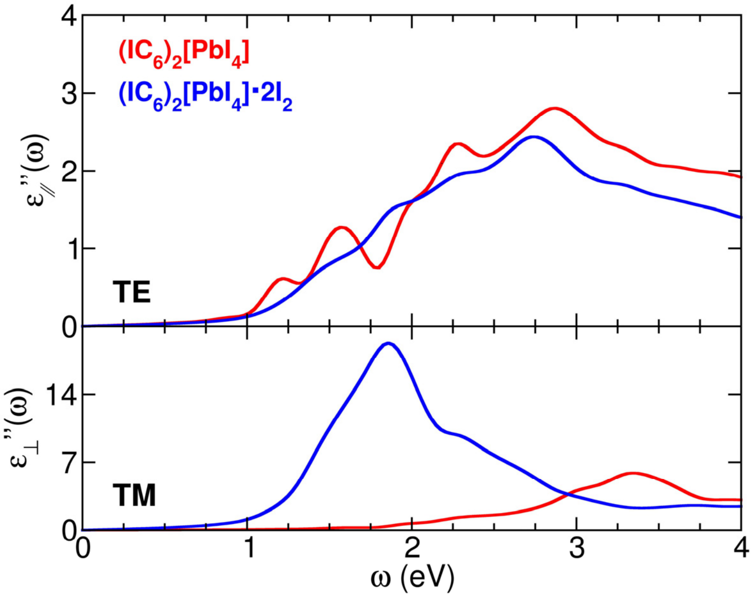

Figure S4. Calculated (at the GGA+vdW+SOC level of theory) absorbance spectra with the direction of light propagation oriented perpendicular to the inorganic sheets ($\varepsilon_{\parallel}''$) and with the direction of light propagation oriented parallel to the inorganic sheets ($\varepsilon_{\perp}''$) as a function of frequency for $(IC_6)_2[PbI_4]$ and $(IC_6)_2[PbI_4]\cdot 2I_2$.

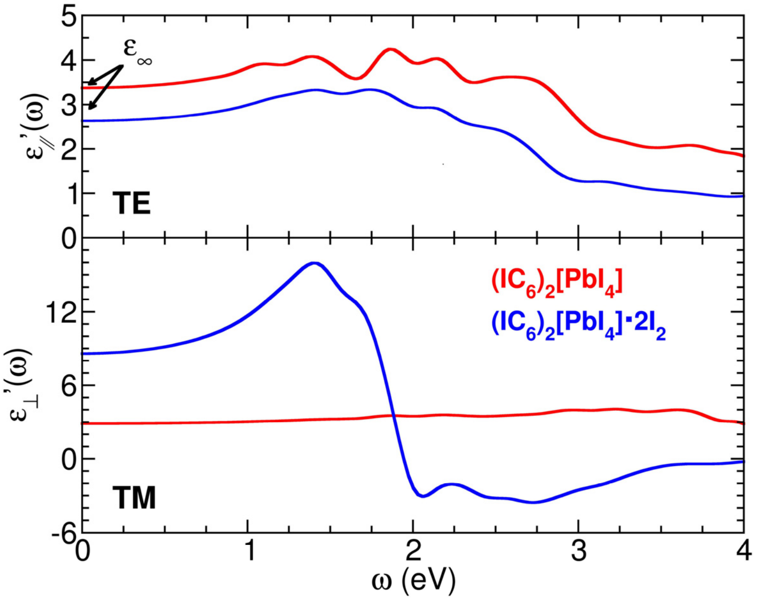

Figure S5. Calculated (at the GGA+vdW+SOC level of theory) real component of the dielectric response parallel ($\varepsilon'_{\parallel}$) and perpendicular ($\varepsilon'_{\perp}$) to the inorganic sheets as a function of frequency for $(IC_6)_2[PbI_4]$ and $(IC_6)_2[PbI_4]\cdot 2I_2$. The high-frequency dielectric constant (ε_{∞}) corresponds to the extrapolation at zero energy.

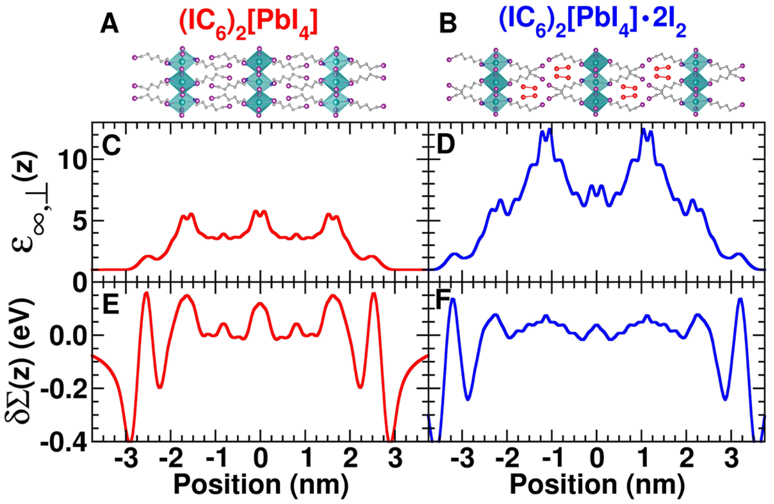

Figure S6. Slabs of (A) $(IC_6)_2[PbI_4]$ and (B) $(IC_6)_2[PbI_4]\cdot 2I_2$ used to compute the high-frequency dielectric profiles $\varepsilon_{\infty,\perp}(z)$, shown respectively in panels (C) and (D), and self-energy profiles $\delta\Sigma(z)$ shown respectively in panels (E) and (F), along the [001] direction.

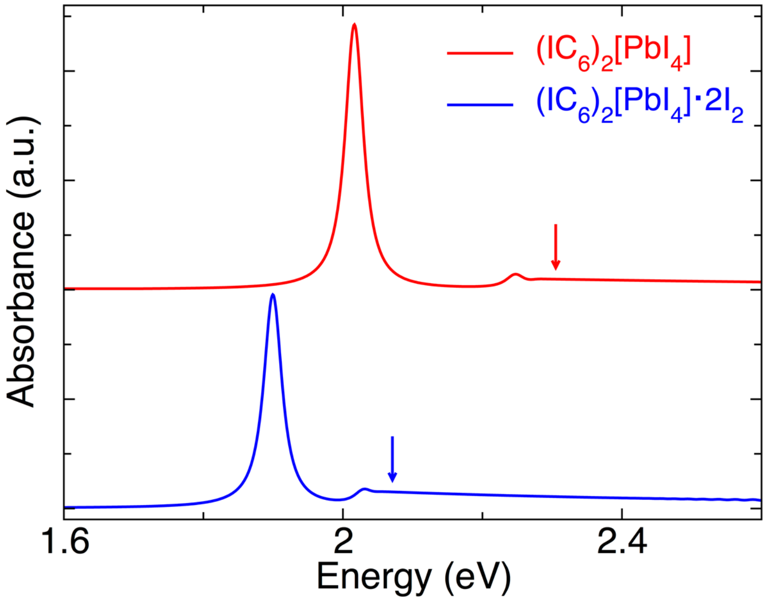

Figure S7. Computed absorbance spectra for $(IC_6)_2[PbI_4]$ and $(IC_6)_2[PbI_4]{\cdot}2I_2$ using the Bethe-Salpeter equation and the self-energy-corrected DFT bandgaps. Arrows indicate the bandgap including self-energy corrections and beginning of the continuum absorption.

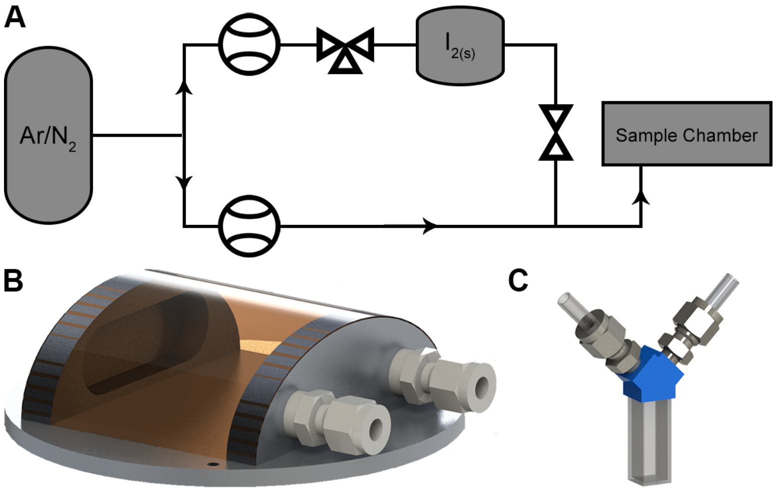

Figure S8. Flow diagram for in situ PXRD and optical absorbance measurements (A). Images of the PXRD (B) and optical absorbance (C) cells.

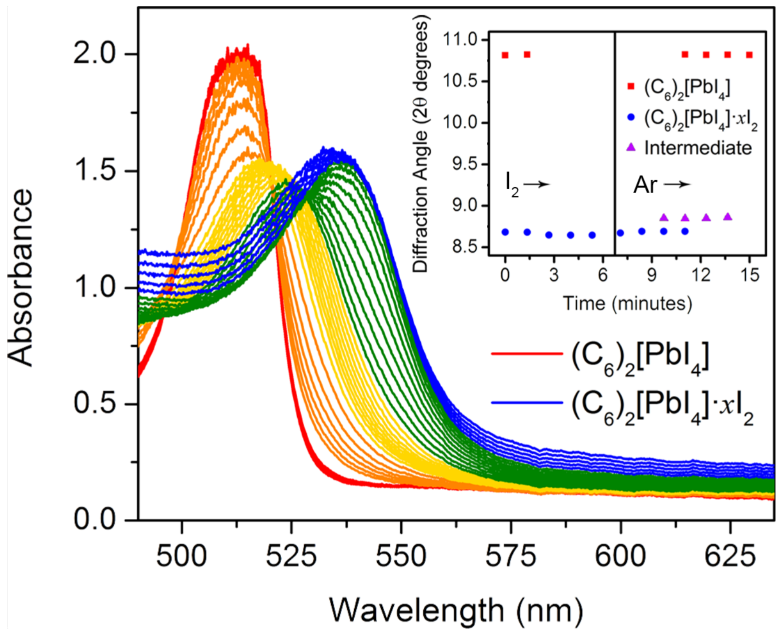

Figure S9. In situ optical absorbance spectra acquired as $(C_6)_2[PbI_4]$ (red) absorbs I_2 to yield $(C_6)_2[PbI_4]\cdot xI_2$ (blue). Inset: Position of the (004) reflection in the powder X-ray diffraction pattern as $(C_6)_2[PbI_4]$ absorbs and then desorbs I_2 vapor.

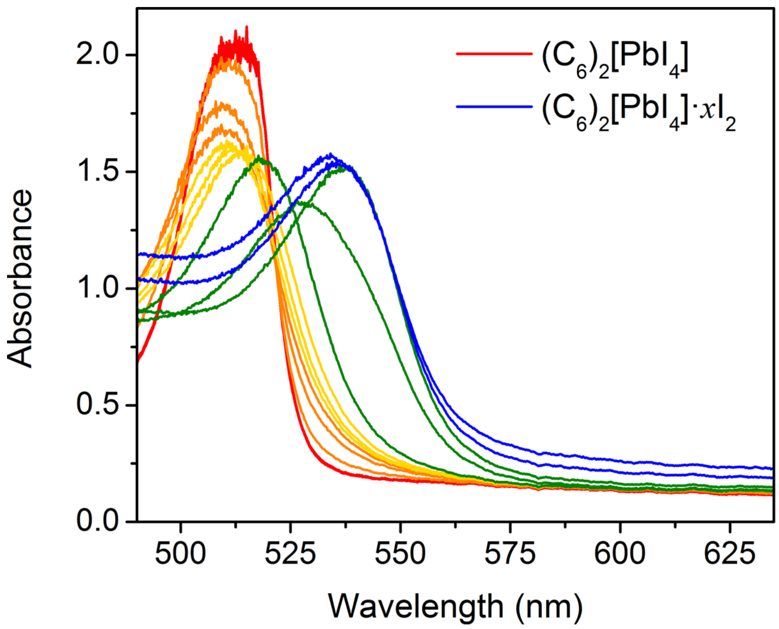

Figure S10. In situ optical absorbance spectra of $(C_6)_2[PbI_4]\cdot xI_2$ (blue) as it desorbs I_2 to form $(C_6)_2[PbI_4]$ (red) under a flow of N_2 gas.

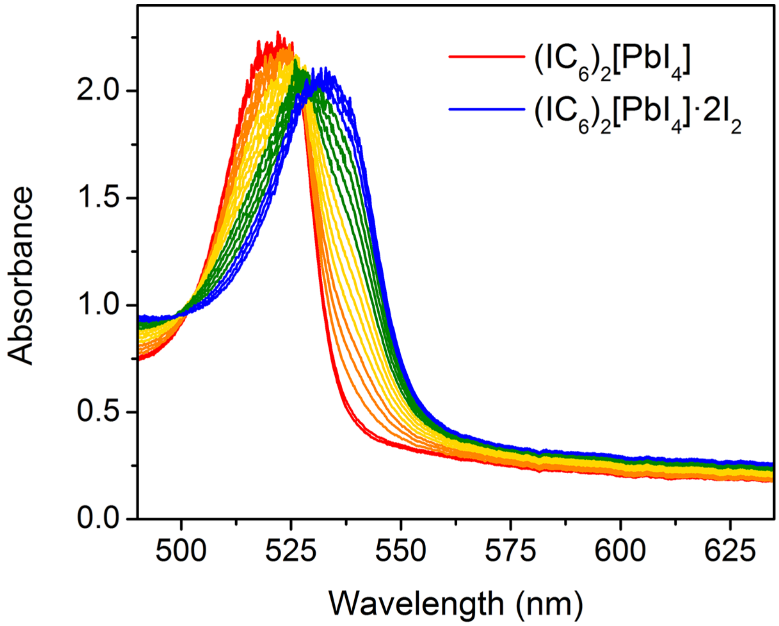

Figure S11. In situ optical absorbance spectra of $(IC_6)_2[PbI_4]$ (red) as it transforms to $(IC_6)_2[PbI_4]\cdot 2I_2$ (blue) under a flow of I_2 gas.

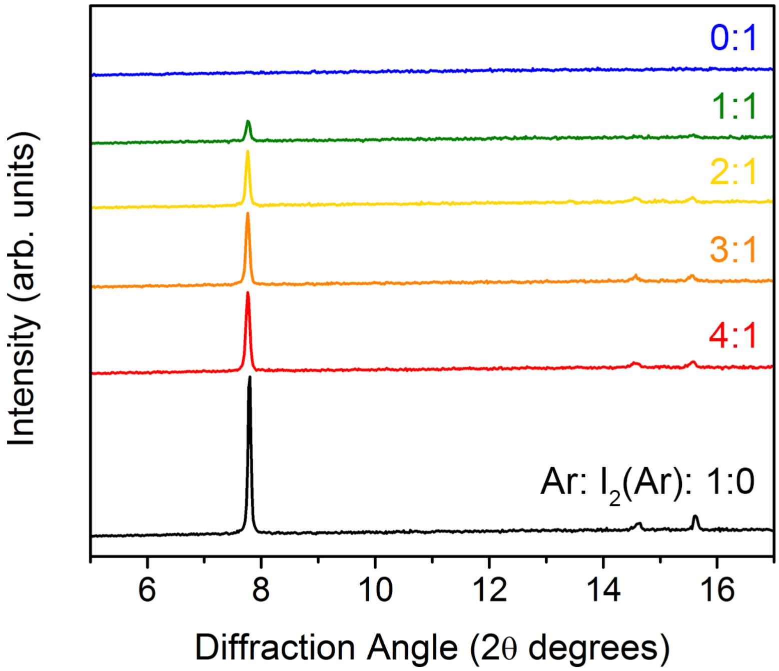

Figure S12. Powder X-ray diffraction patterns of $(H_3NC_6H_{12}NH_3)[PbI_4]$ (a perovskite containing an organic monolayer, which should not permit I_2 intercalation) as it is successively exposed to increasing concentrations of I_2 vapor in Ar gas. The reflections corresponding to the original material (black) decrease in intensity while no new reflections appear. This indicates that the material becomes amorphous without I_2 intercalation.

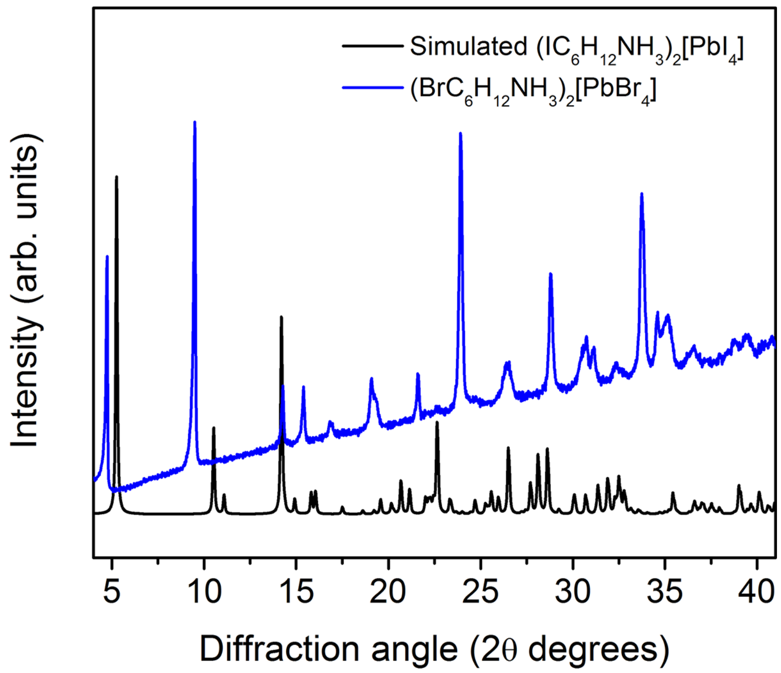

Figure S13. Powder X-ray diffraction pattern of $(BrC_6H_{12}NH_3)_2[PbBr_4]$ (blue) compared to the simulated pattern of $(IC_6H_{12}NH_3)_2[PbI_4]$ (black).

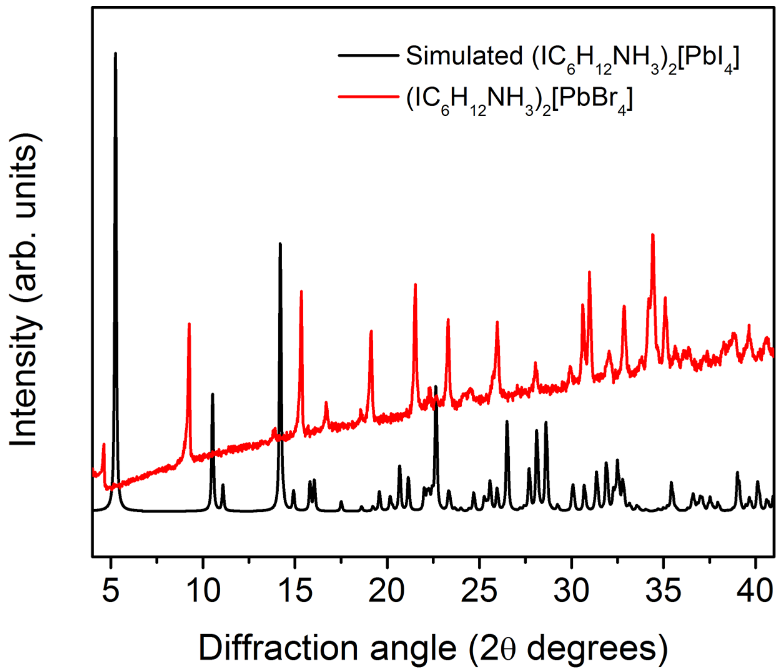

Figure S14. Powder X-ray diffraction pattern of $(IC_6H_{12}NH_3)_2[PbBr_4]$ (red) compared to the simulated pattern of $(IC_6H_{12}NH_3)_2[PbI_4]$ (black).

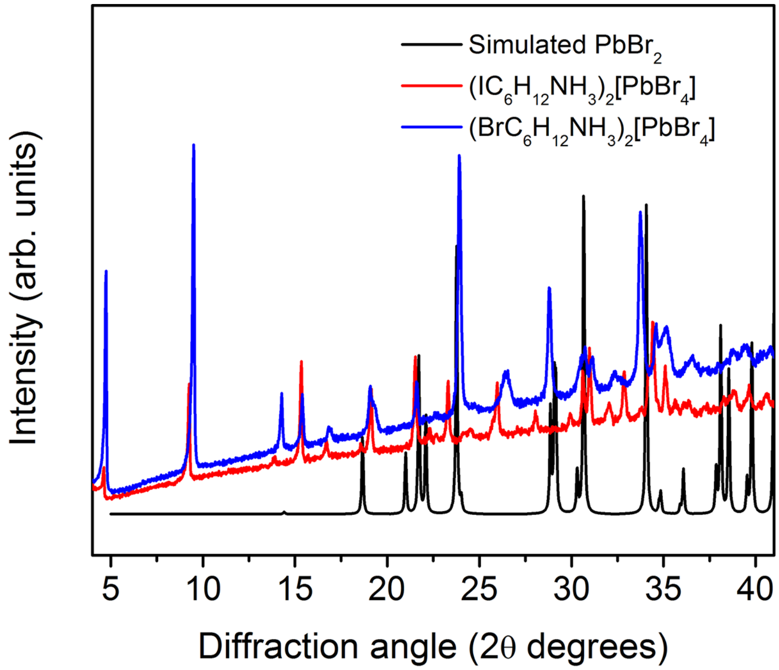

Figure S15. Powder X-ray diffraction patterns of $(IC_6H_{12}NH_3)_2[PbBr_4]$ (red) and $(BrC_6H_{12}NH_3)_2[PbBr_4]$ (blue) compared to the simulated pattern of $PbBr_2$ (black).

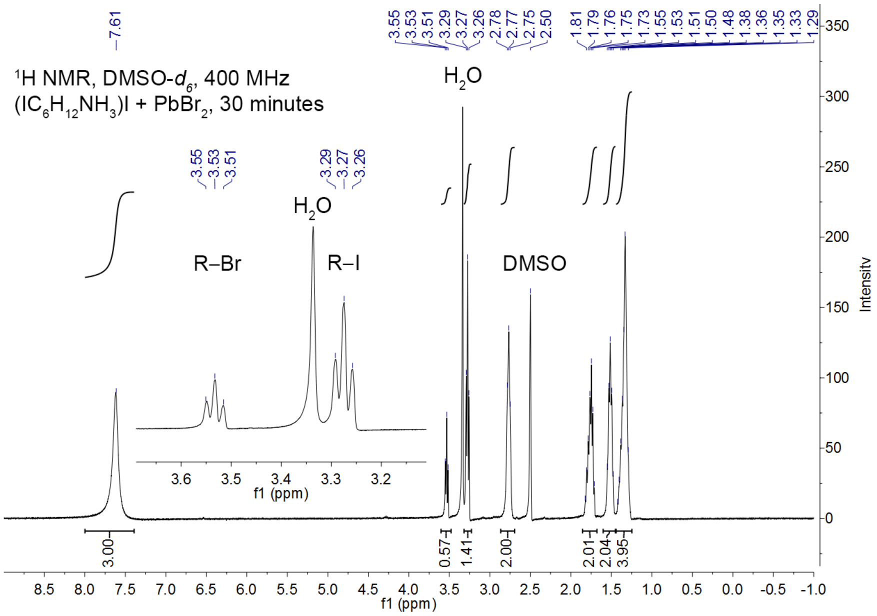

Figure S16. ^{1}H NMR spectrum of $(IC_6H_{12}NH_3)I$ and $PbBr_2$ after 30 minutes in DMSO-*d*$_6$. Inset: Close-up of the spectral region containing primary alkyl-halide resonances. Note the presence of peaks assigned to both terminal alkyl bromide (δ 3.53) and alkyl iodide (δ 3.27) functionalities as the organohalides exchange in solution.

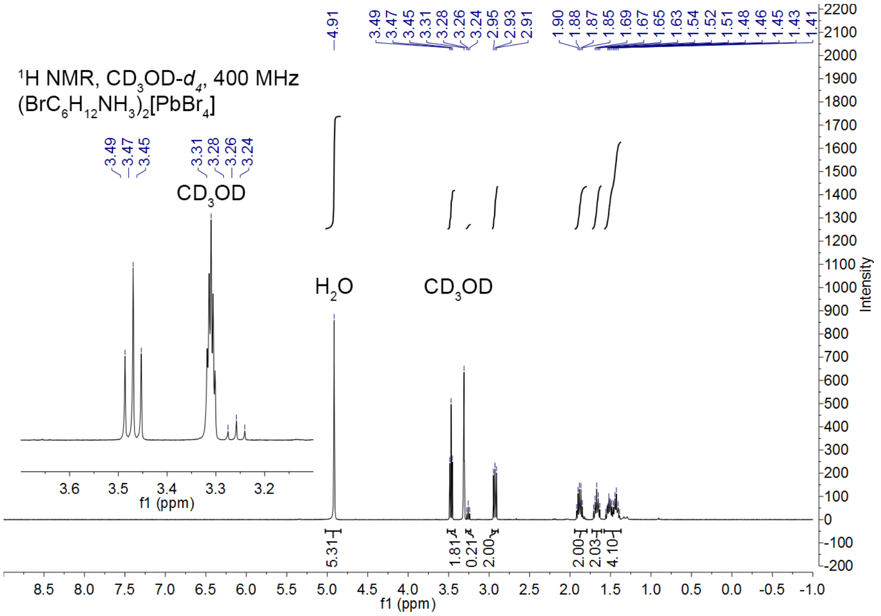

Figure S17. ^{1}H NMR spectrum of $(IC_6H_{12}NH_3)_2[PbI_4]$ following reaction with an excess of Br_2 to form $(BrC_6H_{12}NH_3)_2[PbBr_4]$. Inset: Close-up of the spectral region containing primary alkyl-iodide (δ 3.26) and alkyl-bromide (δ 3.47) resonances. Approximately 10% of the original alkyl iodide remains.

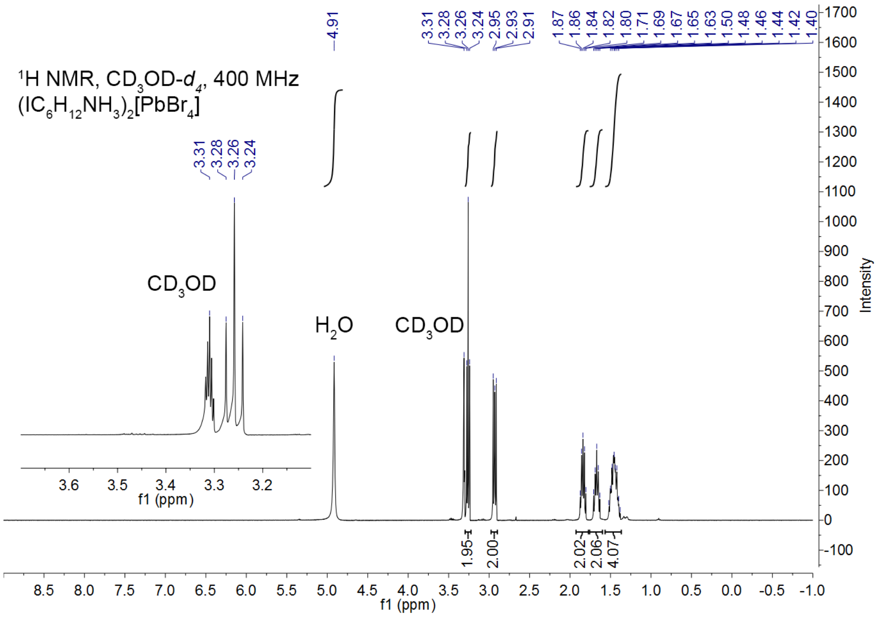

Figure S18. ^{1}H NMR spectrum of $(IC_6H_{12}NH_3)_2[PbBr_4]$. Inset: Close-up of the spectral region containing primary alkyl-iodide resonances (δ 3.26).

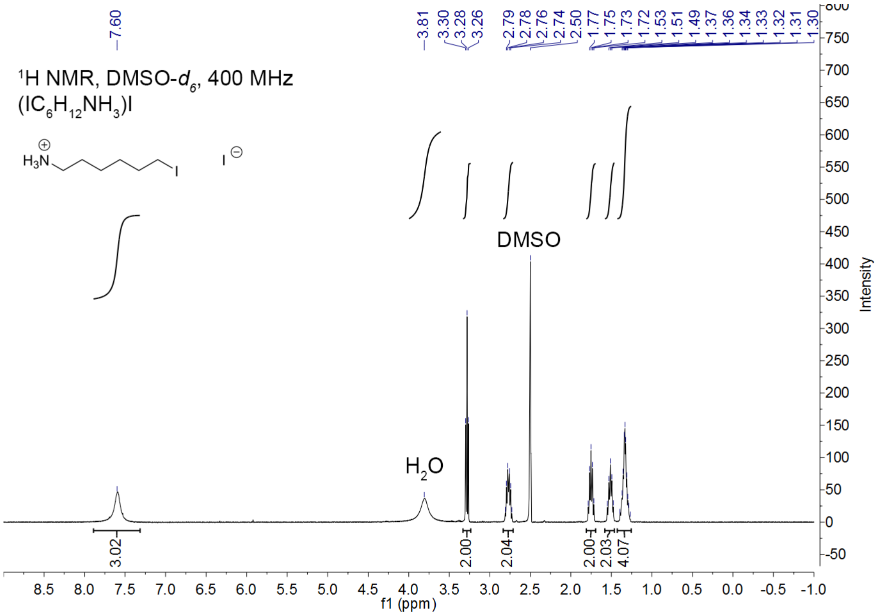

Figure S19. ^{1}H NMR spectrum of $(IC_6H_{12}NH_3)I$.

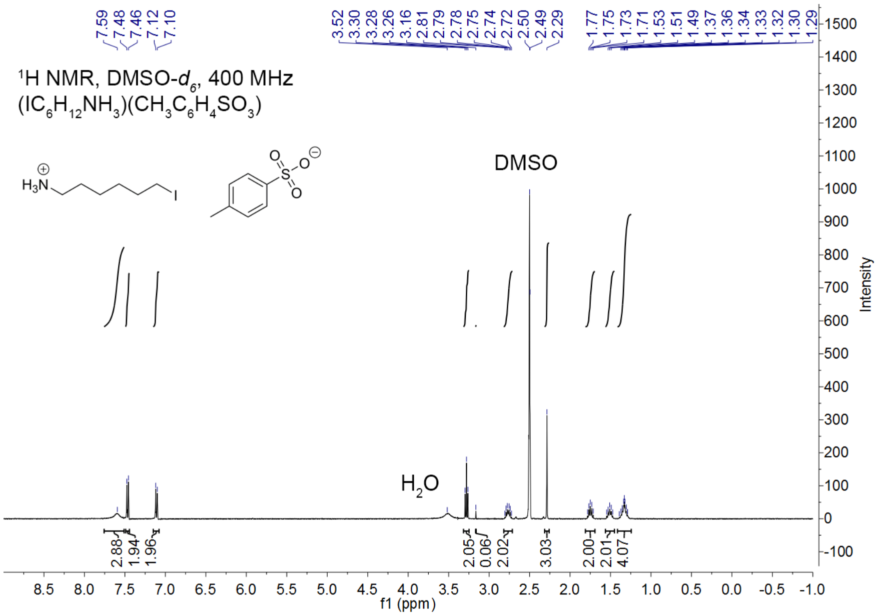

Figure S20. ^{1}H NMR spectrum of $(IC_6H_{12}NH_3)(CH_3C_6H_4SO_3)$.

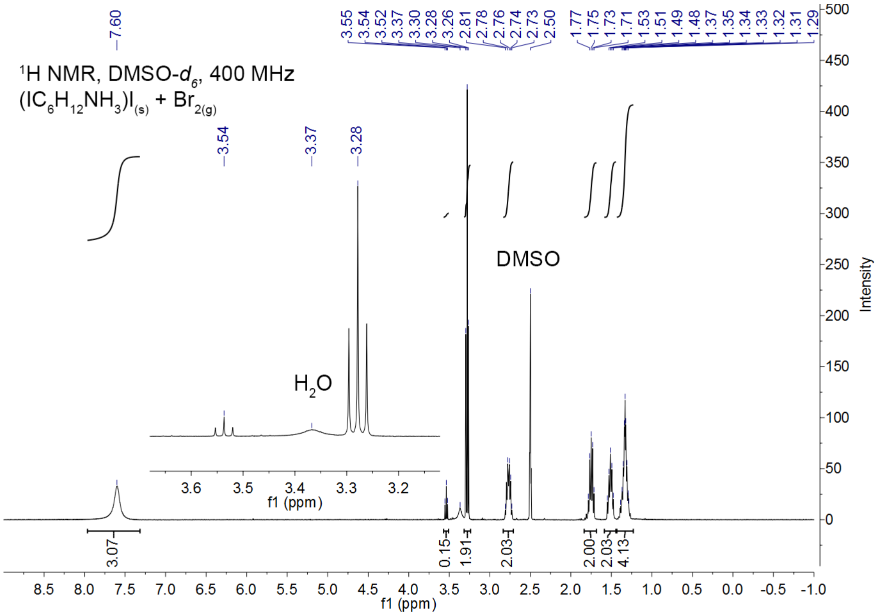

Figure S21. ^{1}H NMR spectrum of the reaction products of $(IC_6H_{12}NH_3)I$ with Br_2. Inset: Close-up of the spectral region containing primary alkyl-halide resonances. Note the presence of peaks assigned to both terminal alkyl bromide (δ 3.54) and alkyl iodide (δ 3.28) functionalities. Only a small amount of the alkyl bromide product is present.

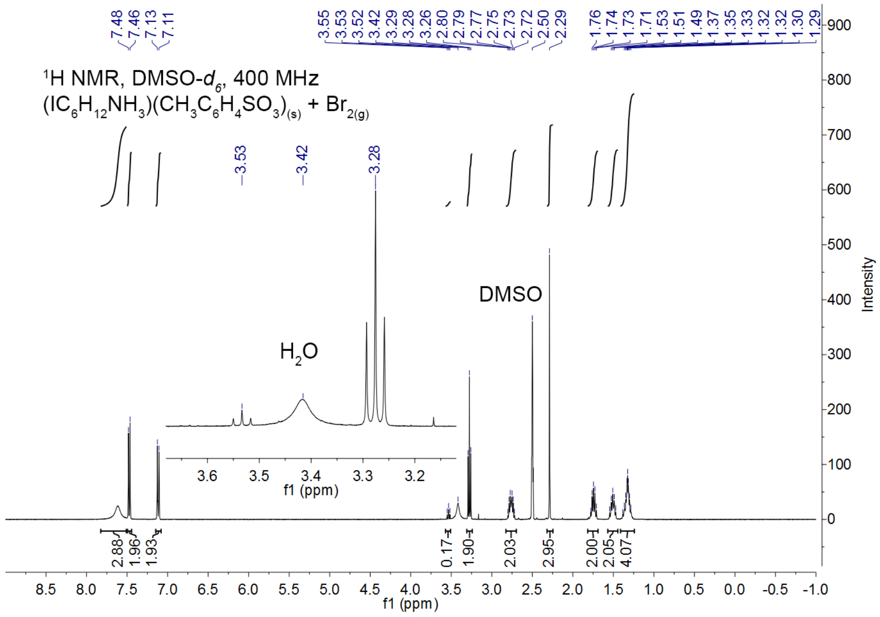

Figure S22. ^{1}H NMR spectrum of the reaction products of $(IC_6H_{12}NH_3)(CH_3C_6H_4SO_3)$ with Br_2. Inset: Close-up of the spectral region containing primary alkyl-halide resonances. Note the presence of peaks assigned to both terminal alkyl bromide (δ 3.53) and alkyl iodide (δ 3.28) functionalities. Only a small amount of the alkyl bromide product is present.

References

1. S. M. A. Rahman, T. Baba, T. Kodama, M. A. Islam and S. Obika, *Bioorg. Med. Chem.*, 2012, **20**, 4098-4102.
2. A. Lemmerer and D. G. Billing, *CrystEngComm*, 2010, **12**, 1290-1301.
3. D. Solis-Ibarra, I. C. Smith and H. I. Karunadasa, *Chem. Sci.*, 2015, **6**, 4054-4059.
4. *SAINT and SADABS*, Bruker AXS Inc.: Madison, Wisconsin, 2007.
5. G. M. Sheldrick, *Acta Crystallogr., Sect. A: Found. Crystallogr.*, 2008, **64**, 112-122.
6. G. M. Sheldrick, *SHELXL-97, A program for crystal structure refinement*, Göttingen, 1997.
7. O. V. Dolomanov, L. J. Bourhis, R. J. Gildea, J. A. K. Howard and H. Puschmann, *J. Appl. Crystallogr.*, 2009, **42**, 339-341.
8. P. Müller, R. Herbst-Irmer, A. L. Spek, T. R. Schneider and M. R. Sawaya, *Crystal Structure Refinement: A Crystallographer's Guide to SHELXL*, Oxford University Press, New York, 2006.
9. P. E. Blöchl, *Phys. Rev. B: Condens. Matter Mater. Phys.*, 1994, **50**, 17953-17979.
10. G. Kresse and D. Joubert, *Phys. Rev. B: Condens. Matter Mater. Phys.*, 1999, **59**, 1758-1775.
11. G. Kresse and J. Furthmüller, *Comput. Mater. Sci.*, 1996, **6**, 15-50.
12. G. Kresse and J. Furthmüller, *Phys. Rev. B: Condens. Matter Mater. Phys.*, 1996, **54**, 11169-11186.
13. M. Shishkin and G. Kresse, *Phys. Rev. B: Condens. Matter Mater. Phys.*, 2006, **74**, 035101.
14. M. Dion, H. Rydberg, E. Schröder, D. C. Langreth and B. I. Lundqvist, *Phys. Rev. Lett.*, 2004, **92**, 246401.
15. J. Klimeš, D. R. Bowler and A. Michaelides, *J. Phys.: Condens. Matter*, 2010, **22**, 022201.
16. J. Klimeš, D. R. Bowler and A. Michaelides, *Phys. Rev. B: Condens. Matter Mater. Phys.*, 2011, **83**, 195131.
17. J. Heyd, G. E. Scuseria and M. Ernzerhof, *J. Chem. Phys.*, 2003, **118**, 8207-8215.
18. J. Heyd, G. E. Scuseria and M. Ernzerhof, *J. Chem. Phys.*, 2006, **124**, 219906.
19. J. Paier, M. Marsman, K. Hummer, G. Kresse, I. C. Gerber and J. G. Ángyán, *J. Chem. Phys.*, 2006, **124**, 154709.
20. H. J. Monkhorst and J. D. Pack, *Phys. Rev. B: Solid State*, 1976, **13**, 5188-5192.
21. J. D. Pack and H. J. Monkhorst, *Phys. Rev. B: Solid State*, 1977, **16**, 1748-1749.
22. J. M. Soler, E. Artacho, J. D. Gale, A. García, J. Junquera, P. Ordejón and D. Sánchez-Portal, *J. Phys.: Condens. Matter*, 2002, **14**, 2745-2779.
23. E. Artacho, E. Anglada, O. Diéguez, J. D. Gale, A. García, J. Junquera, R. M. Martin, P. Ordejón, J. M. Pruneda, D. Sánchez-Portal and J. M. Soler, *J. Phys.: Condens. Matter*, 2008, **20**, 064208.
24. N. Troullier and J. L. Martins, *Phys. Rev. B: Condens. Matter Mater. Phys.*, 1991, **43**, 1993-2006.
25. E. Artacho, D. Sánchez-Portal, P. Ordejón, A. García and J. M. Soler, *Phys. Status Solidi B*, 1999, **215**, 809-817.
26. L. Fernández-Seivane, M. A. Oliveira, S. Sanvito and J. Ferrer, *J. Phys.: Condens. Matter*, 2006, **18**, 7999-8013.
27. J. Even, L. Pedesseau and M. Kepenekian, *Phys. Chem. Chem. Phys.*, 2014, **16**, 25182-25190.
28. D. Sapori, M. Kepenekian, L. Pedesseau, C. Katan and J. Even, *Nanoscale*, 2016, **8**, 6369-6378.
29. M. Shinada and S. Sugano, *J. Phys. Soc. Jpn.*, 1966, **21**, 1936-1946.
30. H. Takagi, H. Kunugita and K. Ema, *Phys. Rev. B: Condens. Matter Mater. Phys.*, 2013, **87**, 125421.
31. E. A. Muljarov, S. G. Tikhodeev, N. A. Gippius and T. Ishihara, *Phys. Rev. B: Condens. Matter Mater. Phys.*, 1995, **51**, 14370-14378.
32. K. Tanaka, T. Takahashi, T. Kondo, K. Umeda, K. Ema, T. Umebayashi, K. Asai, K. Uchida and N. Miura, *Jpn. J. Appl. Phys., Part 1*, 2005, **44**, 5923-5932.
33. X. Hong, T. Ishihara and A. V. Nurmikko, *Phys. Rev. B: Condens. Matter Mater. Phys.*, 1992, **45**, 6961-6964.
34. S.-L. Chuang, S. Schmitt-Rink, D. A. B. Miller and D. S. Chemla, *Phys. Rev. B: Condens. Matter Mater. Phys.*, 1991, **43**, 1500-1509.

35. M. Kepenekian, R. Robles, C. Katan, D. Sapori, L. Pedesseau and J. Even, *ACS Nano*, 2015, **9**, 11557-11567.
